# The efficacy, safety, and tolerability of ivermectin compared with current topical treatments for the inflammatory lesions of rosacea: a network meta-analysis

**DOI:** 10.1186/s40064-016-2819-8

**Published:** 2016-07-22

**Authors:** Kashif Siddiqui, Linda Stein Gold, Japinder Gill

**Affiliations:** PAREXEL Access Consulting, PAREXEL International, 3rd Floor, DLF Tower E, Rajiv Gandi IT Park, Chandigarh, UT 160101 India; Department of Dermatology, Henry Ford Medical Centre, Detroit, MI USA

**Keywords:** Papulopustular, Rosacea, Ivermectin, Topical

## Abstract

**Background:**

Rosacea is a common chronic skin condition that manifests as recurrent inflammatory lesions. Long-term treatment is required to control symptoms and disease progression, with topical treatments being the first-line choice. Ivermectin 1 % cream is a new once-daily (QD) topical treatment for the inflammatory lesions of rosacea, and it is important to compare the efficacy, safety, and tolerability of ivermectin with other currently available topical treatments.

**Methods:**

A systematic literature review was performed from January 2011 to June 2015, with articles published prior to 2011 retrieved from a Cochrane review on rosacea. Randomized controlled trials of the topical treatment of adult patients with moderate-to-severe papulopustular rosacea were identified from electronic databases and trial registers, and supplemented with data from clinical study reports. Mixed treatment comparisons (MTCs) were conducted to compare different treatments according to Bayesian methodology.

**Results:**

57 studies were identified, with 19 providing data suitable for MTC. Ivermectin 1 % cream QD led to a significantly greater likelihood of success compared with azelaic acid 15 % gel twice-daily (BID) [relative risk (95 % credible interval): 1.25 (1.14–1.37)], and metronidazole 0.75 % cream BID [1.17 (1.08–1.29)] at 12 weeks. Ivermectin 1 % cream QD also demonstrated a significant reduction in inflammatory lesion count compared with azelaic acid 15 % gel BID [−8.04 (−12.69 to −3.43)] and metronidazole 0.75 % cream BID [−9.92 (−13.58 to −6.35)] at 12 weeks. Ivermectin 1 % cream QD led to a significantly lower risk of developing any AE or TRAE compared with azelaic acid 15 % gel BID [0.83 (0.71–0.97) and 0.47 (0.32–0.67), respectively].

**Conclusions:**

Ivermectin 1 % cream QD appears to be a more effective topical treatment than other current options for the inflammatory lesions of rosacea, with at least an equivalent safety and tolerability profile, and could provide physicians and dermatologists with an alternative first-line treatment option.

**Electronic supplementary material:**

The online version of this article (doi:10.1186/s40064-016-2819-8) contains supplementary material, which is available to authorized users.

## Background

Papulopustular rosacea is a common chronic skin disease that affects the central facial area, primarily manifesting as recurrent inflammatory episodes of papules and/or pustules and persistent erythema (Cribier 2013), with secondary manifestations including stinging, burning, and flushing (Goldgar et al. 2009). Rosacea is more prevalent in fair-skinned people, affecting approximately 10 % of the Caucasian population, but has also been reported in people of other ethnicities and can affect people of many skin types (Huynh 2013). Overall, it is estimated that 16 million people are affected with rosacea in the United States (Maier 2011), with 40 million people being affected worldwide (Moore 2015).

Although there is no increase in mortality with rosacea, the chronic nature of the disease and expression of symptoms in the facial region may lead to stigmatization. As a result, it is associated with a significant adverse impact on quality of life (QoL) (Goldgar et al. 2009; Aksoy et al. 2010; Wolf and Del Rosso 2007) and may lead to depression or social anxiety disorder (Bohm et al. 2014). The stigma attached to this disease has been confirmed by a recent Global Perception survey, in which subjects with facial redness were judged more negatively than those without redness (Moore 2015). In addition to the psychological burden, the chronic and progressive nature of papulopustular rosacea may disrupt everyday life and work, with onset generally occurring between the ages of 30 years and 50 years (Powell 2005; Moore 2015) and more commonly in females than males (Culp and Scheinfeld 2009).

As with most chronic skin diseases, papulopustular rosacea is treatable rather than curable and requires long-term intervention to control symptoms and prevent disease progression. Topical treatments are the first-line choice for patients due to a lower risk of adverse events (AEs), drug interactions, and antibiotic resistance compared with systemic therapy (Goldgar et al. 2009). A range of topical formulations are currently available to treat papulopustular rosacea (commonly azelaic acid or metronidazole), and there is continued debate over which interventions are the safest and most effective for treating patients (Elewski et al. 2011). In addition, a new topical agent, ivermectin 1 % cream once daily (QD; SOOLANTRA^®^), is now available for the treatment of the inflammatory lesions of rosacea, approved by the US Food and Drug Administration in December 2014 (FDA. 2014; Galderma 2015a) and recently via the decentralized procedure in Europe (Galderma 2015b). With the introduction of new therapies such as ivermectin 1 % cream QD, it is important to understand which treatment can provide patients with the greatest clinical benefit.

A previous systematic review by the Cochrane Collaboration searched the literature up to February 9 2011, identifying 58 trials providing evidence to support the use of metronidazole and azelaic acid in the treatment of rosacea (of which a majority of patients had papulopustular rosacea). However, from this data it remained unclear which of these two treatments was the most effective (van Zuuren et al. 2011). The Cochrane Collaboration systematic review has now been updated (van Zuuren et al. 2015), with the aim of determining the most effective strategy for the treatment of rosacea. The 2015 Cochrane Collaboration review found evidence to support the use of topical azelaic acid, metronidazole, ivermectin, brimonidine, oral doxycycline, and oral tetracycline in the treatment of rosacea through the pooling of direct head-to-head comparison data, typically versus placebo/vehicle (van Zuuren et al. 2015). However, the focus was on the meta-analysis of direct data and as such a network meta-analysis (NMA) utilizing indirect comparison was not planned within the methodology. This means that the majority of pooled evidence compares an active treatment to placebo/vehicle, and cannot provide a comparison between different active treatments.

In order to aid treatment choice for patients with papulopustular rosacea, it is important to compare treatments to understand their relative efficacy, safety, and tolerability profiles. The aim of this review was to quantitatively compare the clinical benefit of ivermectin 1 % cream QD with other current topical treatment options. Ivermectin 1 % cream QD was the focus for the comparison since this is the only new treatment for the inflammatory lesions of rosacea to become available for several years. Although head-to-head data are available compared with metronidazole 0.75 % cream BID (Taieb et al. 2015a), it is of interest to compare ivermectin 1 % cream QD with all currently available topical therapies. This study therefore expands and builds upon the results of the systematic literature reviews conducted in 2011 (van Zuuren et al. 2011) and 2015 (van Zuuren et al. 2015), focusing specifically on patients with papulopustular rosacea, and using the data identified to compare ivermectin 1 % cream QD to the currently available therapy options for these patients through a NMA.

## Methods

A systematic review was initially conducted from January 2011 to June 2014 to update the evidence provided by the earlier Cochrane review relating to the topical treatment of papulopustular rosacea, using the same methodology except where indicated (van Zuuren et al. 2011). Given the publication of the most recent Cochrane review (van Zuuren et al. 2015), the systematic review was updated to June 15 2015 to ensure all the relevant data were identified. A NMA was then conducted to evaluate the efficacy, safety, and tolerability of ivermectin 1 % cream QD against currently available topical treatment options for papulopustular rosacea.

### Data sources

The electronic database searches were originally conducted from January 2011 to June 19 2014 and were then updated for the period June 2014 to June 15 2015. The same search strategy was used for the original systematic review and the systematic review update; an example search strategy is presented in the Additional file 1: Table 1. Bibliographic screening of relevant published reviews was conducted and trial registers were also searched in both the original systematic review (2014) and the systematic review update (2015). Conference proceedings were not searched, in line with the methodology of the Cochrane review. Data from clinical study reports (CSRs) relating to ivermectin 1 % cream QD were provided (Galderma, data on file); the manufacturers of other included comparators were not contacted.

### Study eligibility

The systematic review was conducted in line with the requirements of the Preferred Reporting Items for Systematic Reviews and Meta-Analyses (PRISMA) statement (Moher et al. 2009); studies were included based on pre-defined eligibility criteria.

The studies of interest were randomized controlled trials (RCTs) published in English. Studies reporting on any intervention that might be considered to treat moderate-to-severe papulopustular rosacea, as assessed by individual study investigators, were included in this review. The inclusion of the moderate-to-severe population is in line with the eligibility criteria for the clinical trials of ivermectin and the previous Cochrane Collaboration reviews (van Zuuren et al. 2011, 2015). The interventions included in the review were: ivermectin 1 % cream (SOOLANTRA^®^), azelaic acid 15 % gel (Finacea^®^/Skinoren^®^/Azelex^®^), azelaic acid 20 % cream (Skinoren^®^/Azelex^®^), metronidazole 1 % gel/cream/lotion (Rozex^®^/Metrogel^®^), metronidazole 0.75 % gel/cream/lotion (Rozex^®^/Metrogel^®^), oral antibiotics, pimecrolimus 1 % cream twice daily (BID; Elidel^®^), silica encapsulated benzoyl peroxide with or without topical antibiotics, and sulfacetamide in combination with sulfur.

The patient population of interest was adults (>19 years of age) of any gender or race who had been diagnosed with moderate-to-severe papulopustular rosacea. Studies that included children and adults but did not provide adult subgroup analysis, or that included patients with papulopustular rosacea but did not provide data on moderate-to-severe populations, or enrolled <80 % of patients with moderate-to-severe papulopustular rosacea with no subgroup analysis, were excluded.

### Study selection

The bibliographic details and abstracts of all citations detected through the database, bibliographic, and registry searches were downloaded into the HERON Systematic Review Database. A team of reviewers (information scientists specializing in evidence-based medicine) independently determined the eligibility of each citation by applying the defined eligibility criteria to each title and abstract in a “first-pass” of the studies. The eligibility criteria were then applied to the full-text publications, in a “second-pass” of the studies. Screening was conducted by two independent reviewers, followed by reconciliation by a third independent reviewer.

### Data extraction

One extraction dataset was compiled per study, with multiple publications describing the same study compiled into a single entry to avoid the error of double-counting patients in subsequent analyses. Data was extracted by two independent reviewers from the eligible publications in parallel; a third reviewer subsequently validated the data extraction and resolved any discrepancies.

The outcomes of interest were efficacy (success rate, percentage change in inflammatory lesion count), safety (incidence of any AE, any serious AE [SAE], any treatment-related AE [TRAE], burning/stinging, skin irritation, worsening of erythema, and worsening of rosacea), and tolerability (all-cause withdrawals, withdrawals due to AE). Success rate was defined as either an Investigator Global Assessment (IGA) score of 0 (clear) or 1 (minimal) on a 5-point scale, or 0 (clear), 1 (minimal), or 2 (mild) on a 7-point Likert scale. Sensitivity analyses were performed to analyze the effect of variability in endpoint definition. Percentage change in inflammatory lesion count was defined as the percentage reduction compared to placebo [% reduction = (inflammatory lesion count_tx_/inflammatory lesion count_pbo_) × 100, % reduction vs. placebo = 100 % − % drop, where _tx_ represents the treatment intervention and _pbo_ represents a placebo treatment).

All included citations were critically appraised for quality of reporting in accordance with the SIGN RCT checklist, comprised of two sections and 14 questions, which was applied to each study (SIGN Checklist 2015). Included studies were also appraised for the adequacy of allocation concealment, with a rating from ‘adequate’ to ‘not used’.

### Quantitative data synthesis

Mixed treatment comparisons (MTCs), a form of NMA that combines direct and indirect evidence to synthesize a greater share of the available evidence, were conducted to compare different treatments according to the Bayesian methodology, as recommended by the National Institute for Health and Care Excellence (NICE 2014){Dias, 2013 130/id}. Network diagrams were prepared to identify the MTCs that could be conducted with the available data. MTC analysis was performed using WinBUGS v1.4.3.

Both fixed-effects and random-effects models were used for quantitative analysis. To determine whether a random-effects or fixed-effects model would be most appropriate for this analysis, diagnostics were run based on deviance information criteria (DIC) using both vague priors and informative priors. Convergence of the models was tested over 70,000 iterations with an initial 10,000 burn-ins. The analysis demonstrated Brooks–Gelman–Rubin (BGR) ratios that were close to one, and chains that were well mixed indicating proper convergence of the models. The DIC values were comparable (or lower if the difference is ≥3 points) for the fixed-effects models for all outcomes except for the incidence of burning/stinging. In addition, the credible intervals (CrI) were found to be wide for the random-effects model. With a limited number of studies included in the evidence network, the wider credible intervals might be overwhelmed by the use of vague priors. To assess the impact of priors, a sensitivity analysis using informative priors (suggested by Turney et al.) was conducted. The results of informative priors for the heterogeneity parameter suggested concordance with the fixed effects model. Also, examination of the posterior estimated with actual trial results found the fixed-effects model to be more aligned for all treatment comparisons. As such, only the fixed-effects results are presented; the random effects results are available in the Additional file 1: Tables 2, 3, alongside the DIC results and the comparison of heterogeneity using vague and informative priors (Additional file 1: Tables 4, 5, Fig. 5).

For the dichotomous outcomes (success rate, safety, and tolerability), the results are presented in terms of risk ratios (RR) with 95 % Crl. The number needed-to-treat (NNT) results are also provided for success rate analyses. For the continuous outcome (percentage change in inflammatory lesion count), the results are presented in terms of absolute difference with 95 % Crl. The percentage change in inflammatory lesion count was extracted from the included studies as the mean and standard deviation (SD). In one study by Leyden et al., the mean and SD were highly skewed. Therefore, in order to have symmetry in the data distribution, the median (SD) values were used from this study (Leyden 2014).

In order to capture all data available, time point ranges were defined for the purpose of data analysis. Outcomes are presented at 3 weeks (2–4 weeks or 0.5 months), 6 weeks (5–7 weeks or 1.0 month), 9 weeks (8–10 weeks or 2.0 months), 12 weeks (11–13 weeks or 3.0 months), and 15 weeks (14–16 weeks or 3.5–4.0 months). Success rate and percentage change in inflammatory lesion count were evaluated in a sufficient number of trials to perform a MTC at all time points except at 6 weeks. A lack of relevant studies at this time point led to qualitative analyses being performed for both outcomes. Only 12 week data are presented here; all other time point results are available in the Additional file 1: Tables 7, 8. Safety and tolerability was analyzed by MTC at the 12-week time point only.

## Results

### Study flow

**Fig. 1:**
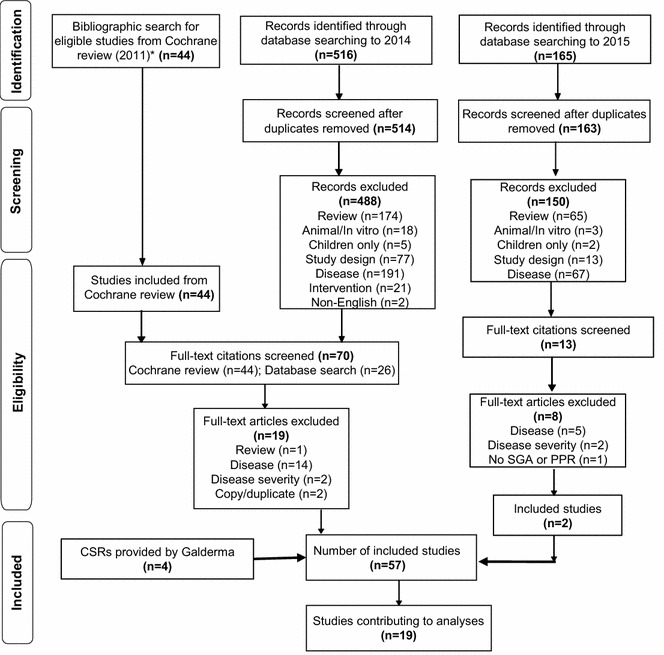
Flow diagram for the systematic review of the efficacy, safety, and tolerability of ivermectin 1 % cream QD compared with current topical treatments for the inflammatory lesions of rosacea. *CSR* clinical study report, *Embase* ^*®*^ Excerpta Medica Database, *MEDLINE* ^*®*^ Medical Literature Analysis and Retrieval System Online, *n* number. *Source: van Zuuren et al. (2011)

Following the original database search to 2014, seven studies were identified in addition to the 44 RCTs identified by the 2011 Cochrane review (van Zuuren et al. 2011) that met the inclusion and exclusion criteria of this review. Four CSRs were provided by Galderma, giving a total of 55 studies included for extraction. Following the systematic review update to June 15, 2015, 57 studies evaluating patients with moderate-to-severe papulopustular rosacea were included overall (N = 10,888). Of these 57 included studies, 19 provided data on one or more of the endpoints of interest that could be quantitatively analyzed [N = 7558 (69 %); Fig. 1]. Compared to the Cochrane review, there was one new study that contributed data to the analyses of success rate, percentage change in inflammatory lesion, safety, and tolerability at 12 weeks (Draelos et al. 2015), and four studies with additional data (Gold et al. 2014a; Galderma 2014a, 2006). Some studies included in the Cochrane review were excluded in this review; since the Cochrane review did not exclude studies based on the subtype of rosacea, whereas the eligibility criterion for this review was papulopustular rosacea only, the main reason for exclusion of studies was disease (i.e. absence of patients with papulopustular rosacea).

The 19 RCTs included in the NMA enrolled between 72 patients (Fowler 2007a) and 1299 patients (Beutner and Calverese 2005) with a study duration between 10 weeks (Beutner and Calverese 2005) and 56 weeks (including a secondary 40-week follow-up period) (Gold et al. 2014a; Stein et al. 2014) (Table 1). The most commonly investigated interventions were metronidazole 0.75 % cream BID and azelaic acid 15 % gel BID; ivermectin 1 % cream QD was assessed in four studies (Gold et al. 2014a; Galderma 2014a, 2006). Baseline patient characteristics were not found to be significantly different across treatment groups in any of the included studies except one, where disease duration at baseline was reported to be significantly higher in the group receiving pimecrolimus 1 % cream BID compared with the group receiving metronidazole 1 % cream BID (p < 0.05) (Koca et al. 2010). This study contributed data suitable for the tolerability analyses only, therefore it is not expected that differences in baseline disease duration would impact on the pooled results.

**Table 1:** Study details for the RCTs included in the quantitative analyses of the efficacy and safety of topical treatments for moderate-to-severe papulopustular rosacea

Study name/publication	Design	Duration (weeks)	Intervention (n)	Analysis outcomes	Author conclusions
18170 Study (Gold et al. 2014a; Stein et al. 2014) Part A (efficacy) and Part B (safety) *Additional data not included in Cochrane review from CSR*	R, DB, AP, MC-I, Phase III	56	Ivermectin 1 % cream QD (451) Azelaic acid 15 % gel BID (210) – Part B only Vehicle (232)	Inflammatory lesion count Success rate Any AE, any TRAE, any SAE Specific AEs: burning/stinging, skin irritation, worsening of rosacea All cause withdrawals Withdrawal due to AE	Ivermectin 1 % cream QD was well-tolerated and safe for papulopustular rosacea, in particular, no notable difference was found between the ivermectin 1 % cream QD and azelaic acid 15 % gel BID. Ivermectin 1 % cream QD resulted in fewer skin-related AEs than with azelaic acid 15 % gel BID and vehicle
18171 study (Gold et al. 2014b; Stein et al. 2014) Part A (efficacy) and Part B (safety) *Additional data not included in Cochrane review from CSR*	R, DB, AP, MC-I, Phase III	56	Ivermectin 1 % cream QD (459) Azelaic acid 15 % gel BID (208)—Part B only Vehicle (229)	Inflammatory lesion count Success rate Any AE, any TRAE, any SAE Specific AEs: burning/stinging, skin irritation All cause withdrawals Withdrawal due to AE	Ivermectin 1 % cream QD was well-tolerated and safe, with less frequent AEs and a statistically significantly greater success rate at 12 weeks compared to azelaic acid 15 % gel BID when used to treat moderate-to-severe papulopustular rosacea
Beutner and Calverese (2005)	R, IB, AP, MC, PU	10	MET 1 % cream QD (553) MET 1 % gel QD (557) Vehicle (189)	Success rate	Metronidazole gel 1 % QD had a higher efficacy rate than its cream formulation and its vehicle and was equally well-tolerated for the treatment of rosacea
Bjerke et al. (1999)	R, DB, PC, MC, PU	13	Azelaic acid 20 % cream BID (76) Vehicle (39)	Specific AEs: burning/stinging, skin irritation Withdrawal due to AE	Azelaic acid 20 % cream BID was effective and well-tolerated with a significantly greater reduction inflammatory lesion count compared to vehicle for the treatment of papulopustular rosacea
Draelos et al. (2013)	R, DB, PC, MC, PU	16	Azelaic acid 15 % foam BID (198) Vehicle (203)	Inflammatory lesion count Success rate Any TRAE Specific AEs: burning/stinging, worsening of erythema, worsening of rosacea All cause withdrawals Withdrawal due to AE	Azelaic acid 15 % foam BID demonstrated a significant advantage over the vehicle in both primary measures of efficacy: therapeutic success rate (p = 0.017) and change in inflammatory lesion count (p = 0.001) for the treatment of papulopustular rosacea
Draelos et al. (2015) *Not included in Cochrane review*	R, DB, PC, MC, Phase III	16	Azelaic acid 15 % foam BID (484) Vehicle (477)	Inflammatory lesion count Success rate Any SAE All cause withdrawals Withdrawal due to AE	This study supported the efficacy and safety of azelaic acid foam in patients with papulopustular rosacea. Azelaic acid 15 % foam demonstrated a statistically significant advantage over vehicle in both primary measures of efficacy success rate and change in inflammatory lesion count
Elewski et al. (2003)	R, IB, AC, MC, PU	15	Azelaic acid 15 % gel BID (124) MET 0.75 % gel BID (127)	Inflammatory lesion count Success rate	Use of azelaic acid 15 % gel for 15 weeks demonstrated significant superiority over using MET 0.75 % gel in improving principal signs of rosacea (inflammatory lesions and erythema)
Fowler (2007a, b)	R, DB, PC, MC, PU	16	MET 1 % gel BID + Vehicle followed by Vehicle (36) MET 1 % gel BID + DOX 40 mg QD followed by DOX 40 mg QD (36)	Inflammatory lesion count	Combination anti-inflammatory dose DOX and MET 1 % gel resulted in a faster reduction of inflammatory lesion count, when calculated at all interim and final data analysis. Anti-inflammatory dose DOX sustained the reduction in lesion count through 16 weeks in patients with mild-to-moderate rosacea
Koca et al. (2010)	R, OL, AC, SC, PU	12	MET 1 % cream bid (24) PIM 1 % cream bid (25)	All cause withdrawals	PIM 1 % cream was equally effective in reducing inflammatory lesion count as MET 1 % cream in the treatment of papulopustular rosacea
Leyden (2014)	R, DB, AP, MC, Phase II	12	Silica encapsulated benzoyl peroxide 1 % gel QD (32) Silica encapsulated benzoyl peroxide 5 % gel QD (30) Vehicle (30)	Success rate	Silica encapsulated benzoyl peroxide 1 % and 5 % gels were superior to vehicle in reducing papulopustular lesions
NCT00617903 (2013)	R, DB, PC, MC, Phase II	12	Azelaic acid 15 % foam BID (41) Vehicle (42)	Inflammatory lesion count Success rate Any AE Specific AEs: worsening of erythema, worsening of rosacea All cause withdrawals Withdrawal due to AE	Authors’ conclusions about the study drug could not be ascertained from the NCT ID from where the trial data were extracted, no publication for the trial could be retrieved
RD.03.SRE.40027 (Galderma 2006) *Additional data not included in Cochrane review from CSR*	R, IB, AP, MC-I, Phase II	12	Ivermectin 0.1 % cream QD (51) Ivermectin 0.3 % cream QD (47) Ivermectin 1 % cream BID (48) Ivermectin 1 % cream QD (52) MET 0.75 % cream BID (48) Vehicle (50)	Inflammatory lesion count Success rate Any AE, any TRAE, any SAE Specific AEs: burning/stinging, skin irritation, worsening of erythema, worsening of rosacea All cause withdrawals Withdrawal due to AE	Both ivermectin 1 % cream QD and BID were effective and safe, with similar efficacy results between the two dosages. Compliance was enhanced by the ivermectin 1 % QD application for the treatment of papulopustular rosacea
RD.03.SPR.40173 (ATTRACT) (Galderma 2014; Taieb et al. 2015a, b) *Additional data not included in Cochrane review from CSR*	R, IB, AC, MC-I, Phase III	52	Ivermectin 1 % cream QD (478) MET 0.75 % cream BID (484)	Inflammatory lesion count Success rate	Ivermectin 1 % cream resulted in a statistically significant delayed and extended remission when compared to MET 0.75 % cream when used to treat papulopustular rosacea
Tan et al. (2002)	R, DB, PC, MC, PU	12	MET 1 % cream BID (61) Vehicle (59)	Inflammatory lesion count Any AE, any TRAE Specific AEs: burning/stinging, worsening of erythema All cause withdrawals Withdrawal due to AE	The combined topical formulation of MET 1 % cream with sunscreen SPF 15 was effective and well tolerated for the treatment of patients with moderate-to-severe rosacea
Thiboutot et al. (2003)	R, DB, PC, MC, Phase III	12	Azelaic acid 15 % gel BID (164) Vehicle (165)	Inflammatory lesion count Success rate Any AE All cause withdrawals Withdrawal due to AE	The results of these two controlled studies demonstrate that azelaic acid 15 % gel, used twice daily, is an efficacious, safe, and well-tolerated topical treatment for moderate papulopustular rosacea
Thiboutot et al. (2003)	R, DB, PC, MC, Phase III	12	Azelaic acid 15 % gel BID (169) Vehicle (166)	Inflammatory lesion count Success rate Any AE All cause withdrawals Withdrawal due to AE	
Thiboutot et al. (2008)	R, DB, DR, MC, PU	12	Azelaic acid 15 % gel BID (47) Azelaic acid 15 % gel QD (45)	Inflammatory lesion count Success rate Any AE, any TRAE	Once-daily azelaic acid 15 % gel can be utilized as a safe, effective, and economical dosing option for the treatment of mild-to-moderate papulopustular rosacea. Once-daily dosing of azelaic acid 15 % gel was well accepted by patients and can offer considerable dosing flexibility and convenience for the patient as well as for the dermatologist
Torok et al. (2005)	R, DB, AC, MC, PU	12	Sodium sulfacetamide 10 % cream BID + sulfur 5 % cream BID (75) MET 0.75 % cream BID (77)	Inflammatory lesion count Any AE, any TRAE All cause withdrawals	In patients without sulfur drug allergies, sodium sulfacetamide 10 % and sulfur 5 % cream with sunscreen offers greater efficacy than MET 0.75 % cream and has the added benefit of sun protection
Wolf et al. (2006)	R, IB, AC, MC, PU	15	Azelaic acid 15 % gel BID (78) MET 1 % gel QD (82)	Success rate	MET 1 % gel and azelaic acid 15 % gel showed similar reductions in inflammatory lesion count and high success rates in both global severity and erythema in patients with moderate rosacea

*AC* active-controlled, *AE* adverse events, *AP* active- and placebo/vehicle-controlled, *BID* twice daily, *DB* double-blind, *DOX* doxycycline, *DR* dose ranging, *IB* investigator blind, *MC* multicenter, *MC-I* multicenter international, *MET* metronidazole, *N* number of patients, *PC* placebo/vehicle-controlled, *PIM* pimecrolimus, *PU* phase unclear, *QD* once-daily, *R* randomized, *SAE* serious adverse event, *SPF* sun protection factor, *TRAE* treatment-related adverse event

Following the quality assessment using the SIGN checklist, all studies contributing data to the quantitative analyses were found to be of high or acceptable quality, with an adequate method of allocation concealment (full quality appraisal available in the Additional file 1: Table 6). Therefore, no further consideration of quality was made when interpreting the results of the quantitative analyses.

It should be noted that the objective of this systematic review and NMA was to compare ivermectin 1 % cream QD with current topical treatment options for the inflammatory lesions of rosacea. Therefore, only the results comparing ivermectin 1 % cream QD with another treatment are discussed further; for completeness, all comparisons across all treatments included in the NMA for success rate are available as matrix tables in the Additional file 1: Tables 9–16.

### Efficacy

#### Success rate

**Fig. 2:**
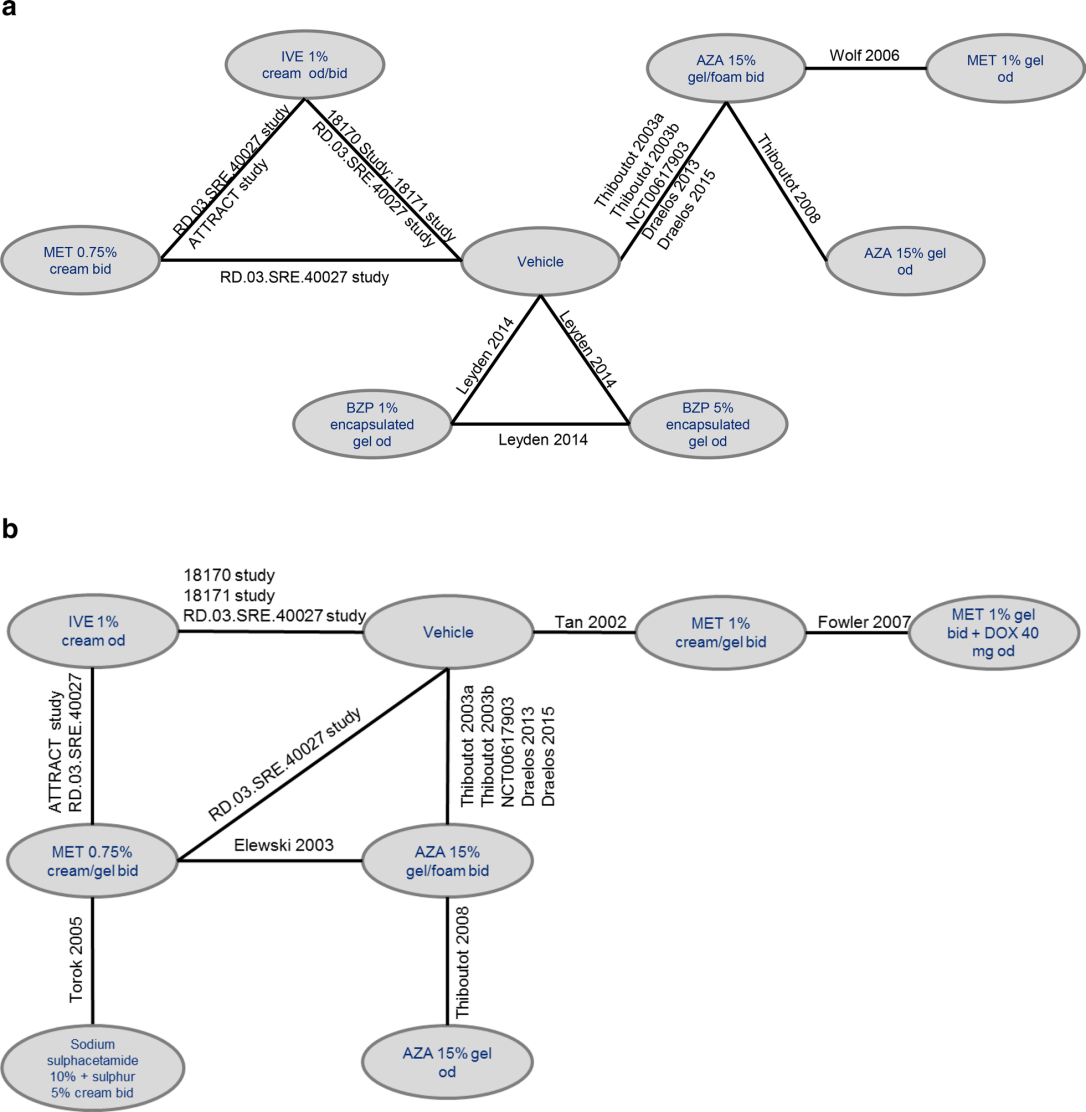
Network diagram for success rate (**a**) and percentage change in inflammatory lesion count (**b**) at 12 weeks. *AZA* azelaic acid, *bid* twice daily, *DOX* doxycycline, *IVE* ivermectin, *MET* metronidazole, *OD* once daily. *Note*: network diagrams for other timepoints are available in the Additional file 1

Success rate was evaluated in 19 of the 57 included studies, with 12 studies contributing data to the quantitative analysis at 12 weeks (Fig. 2a).

The MTC suggested there was a higher likelihood of success with ivermectin 1 % cream QD compared with azelaic acid 15 % gel BID and metronidazole 0.75 % cream BID for up to 12 weeks of treatment, with fewer patients needing to be treated in order for one patient to achieve success (RR analysis of 12 studies; Table 2). Similarly, although only 3 studies were available at the 15 week time point, the analysis also found a significantly greater likelihood of success with ivermectin 1 % cream QD compared to metronidazole 0.75 % gel/cream BID at this time point (Additional file 1: Table 7).

**Table 2:** Results of an MTC of success rate for ivermectin 1 % cream QD versus other available topical treatments and vehicle at 12 weeks

Comparator treatment	12 weeks 12 studies
RR (95 % Crl) (vs. ivermectin 1 % cream QD)	
Azelaic acid 15 % gel QD	1.33 (0.99 to 2.20)
Azelaic acid 15 % gel BID	*1.25 (1.14 to 1.37)*
Metronidazole 0.75 % cream BID	*1.17 (1.08 to 1.29)*
Metronidazole 1 % gel QD	1.18 (0.98 to 1.56)
Silica encapsulated benzoyl peroxide 1 % gel QD	1.09 (0.86 to 1.78)
Silica encapsulated benzoyl peroxide 5 % gel QD	0.94 (0.81 to 1.29)
Vehicle	*1.56 (1.46 to 1.65)*
NNT (95 % Crl) (vs. vehicle)	
Azelaic acid 15 % gel QD	7 (−120 to 120)
Azelaic acid 15 % gel BID	*9 (6 to 14)*
Metronidazole 0.75 % cream BID	*6 (4 to 13)*
Metronidazole 1 % gel QD	*6 (3 to 41)*
Silica encapsulated benzoyl peroxide 1 % gel QD	4 (−31 to 44)
Silica encapsulated benzoyl peroxide 5 % gel QD	*3 (2 to 9)*
Ivermectin 1 % cream QD	*3 (3 to 4)*

Risk ratios evaluate the probability of success (relieving rosacea) when using ivermectin 1 % cream QD, compared to other comparator treatments. A risk ratio >1 demonstrates a greater likelihood of success using ivermectin 1 % cream QD, a RR Crl that does not cross 1 demonstrates a significant difference between ivermectin and the comparator (positive values indicate superiority, negative values indicate inferiority) (indicated in italic). Lower estimates of NNT indicate a greater likelihood of patients achieving success with ivermectin 1 % cream QD, as fewer patients need to be treated to achieve one success than with the comparator treatment. A positive Crl indicates a significantly greater likelihood of patients achieving success when using ivermectin 1 % cream QD than when using the comparator (indicated in italic). Studies contributing to 12 week analysis: Galderma (2006, 2014), Thiboutot et al. (2008), Wolf et al. (2006), NCT00617903 (2013), Draelos et al. (2013, 2015), Stein et al. (2014), Thiboutot et al. (2003), Leyden (2014), Gold et al. (2014a); 3, 9, and 15 weeks results available in Additional file 1: Table 7. MTC results are derived from a fixed effects model. At 15 weeks, ivermectin vs. vehicle data were limited, and so metronidazole 0.75 % BID was used as the bridging comparator for NNT

*BID* twice daily, *Crl* credible interval, *MTC* mixed treatment comparison, *NNT* number needed-to-treat, *QD* once daily, *RR* risk ratio

When comparing ivermectin 1 % cream QD with azelaic acid 15 % gel QD the difference between treatments did not reach statistical significance in the RR analysis, although the NNT was lower with ivermectin 1 % cream QD compared with azelaic acid 15 % gel QD (3 vs. 7). Similarly, ivermectin 1 % cream QD presented comparable results at 12 weeks compared with metronidazole 1 % gel QD, with the NNT values versus vehicle substantially lower for ivermectin 1 % cream QD than for metronidazole 1 % gel QD at 12 weeks (3 vs. 6).

No statistically significant difference was found between silica encapsulated benzoyl peroxide 1 or 5 % gel QD and ivermectin 1 % cream QD at 12 weeks.

As expected, the analysis reported a significantly greater likelihood of treatment success with ivermectin 1 % cream QD compared to vehicle at all time points, both in terms of RR (to 12 weeks) and NNT (to 15 weeks). At 12 weeks, the NNT value of 3 with ivermectin 1 % cream QD against vehicle indicated that as few as three patients needed to be treated with ivermectin 1 % cream QD to achieve one additional success compared to treatment with vehicle. Ivermectin 1 % cream QD was the only active treatment to show statistical superiority to vehicle at all time points investigated and in both analyses (RR and NNT).

The sensitivity analysis of endpoint definition did not identify any differences from the results presented here.

#### Inflammatory lesion count

Inflammatory lesion count was evaluated in 46 of the 57 studies included in the review, with 14 studies providing data for the quantitative analysis at 12 weeks (Fig. 2b). The MTC of ivermectin 1 % cream QD suggested there is a greater percentage reduction in inflammatory lesion count compared to azelaic acid 15 % gel QD and BID, and metronidazole 0.75 % cream BID at 12 weeks (Table 3). No significant differences were observed between ivermectin 1 % cream QD and metronidazole 1 % gel BID or metronidazole 1 % gel BID combined with doxycycline 40 mg QD, or the combination of sodium sulfacetamide 10 % with sulfur 5 % cream BID.

**Table 3:** Results of an MTC of percentage change in inflammatory lesion count between ivermectin 1 % cream QD and comparators at 12 weeks

Comparator treatment	12 weeks (Absolute difference, 95 % Crl) 14 studies
Azelaic acid 15 % gel QD	*−15.87 (−29.02 to −2.87)*
Azelaic acid 15 % gel BID	*−8.04 (−12.69 to −3.43)*
Metronidazole 0.75 % cream BID	*−9.92 (−13.58 to −6.35)*
Metronidazole 1 % gel BID	18.11 (−3.63 to 39.95)
Metronidazole 1 % gel BID + doxycycline 40 mg QD	−0.04 (−25.21 to 25.65)
Sodium sulfacetamide 10 % + sulfur 5 % cream BID	−1.68 (−10.21 to 6.85)
Vehicle	*−21.42 (−25.20 to −17.60)*

Negative values indicate a greater percentage reduction in the inflammatory lesion count with ivermectin 1 % cream QD than with the comparator. A negative Crl that does not cross 0 indicates a significantly higher likelihood of patients using ivermectin 1 % cream QD experiencing a greater reduction in inflammatory lesion count (significant differences between treatments indicated in italic). Studies contributing to 12 week analysis: Stein et al. (2014), Gold et al. (2014a), Galderma (2006, 2014), Torok et al. (2005), Elewski et al. (2003), Thiboutot et al. (2003, 2008), NCT00617903 (2013), Draelos et al. (2013, 2015), Tan et al. (2002), Fowler (2007a); 3 week and 9 week results available in Additional file 1: Table 8. MTC results are derived from a fixed effects model

*BID* twice daily, *Crl* credible interval, *MTC* mixed treatment comparison, *QD* once daily

As observed for success rate, ivermectin 1 % cream QD was associated with a significantly greater reduction in inflammatory lesion count than vehicle at all evaluable time points up to 12 weeks.

A post hoc sensitivity analysis on the impact of inclusion of a Phase II trial (Galderma 2006) was conducted for success rate and percentage change in inflammatory lesion count at 12 weeks. The analysis demonstrated no change in the original analysis results in terms of direction of treatment effect or level of significance.

### Safety

**Fig. 3:**
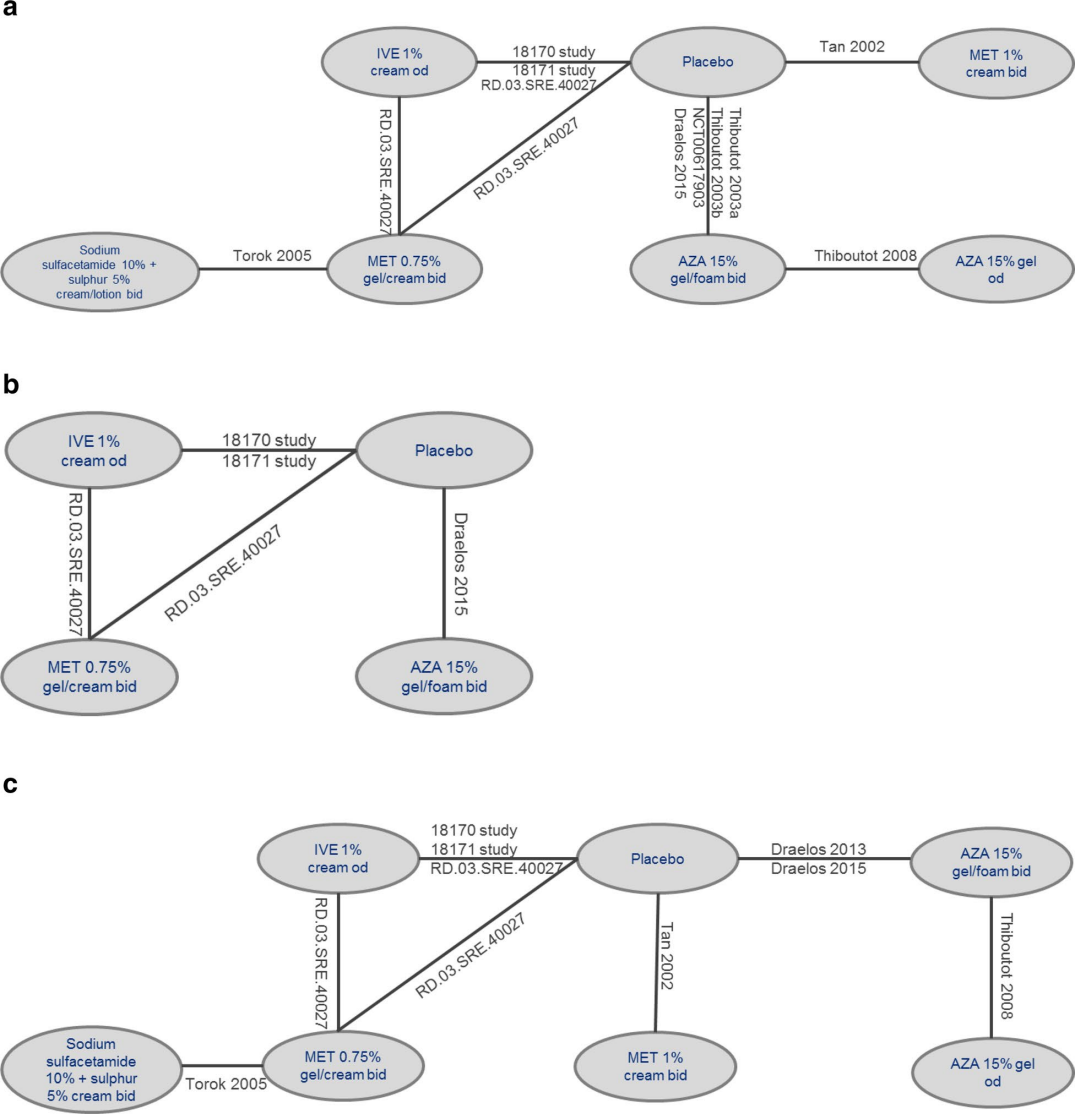
Network diagrams for incidence of any adverse events (**a**), any serious adverse events (**b**), and any treatment-related adverse events (**c**) at 12 weeks. *AZA* azelaic acid, *bid* twice daily, *IVE* ivermectin, *MET* metronidazole, *OD* once daily. *Note*: network diagrams for the incidence of burning/stinging, skin irritation, worsening of erythema, and worsening of rosacea at 12 weeks are available in the Additional file 1

**Fig. 4:**
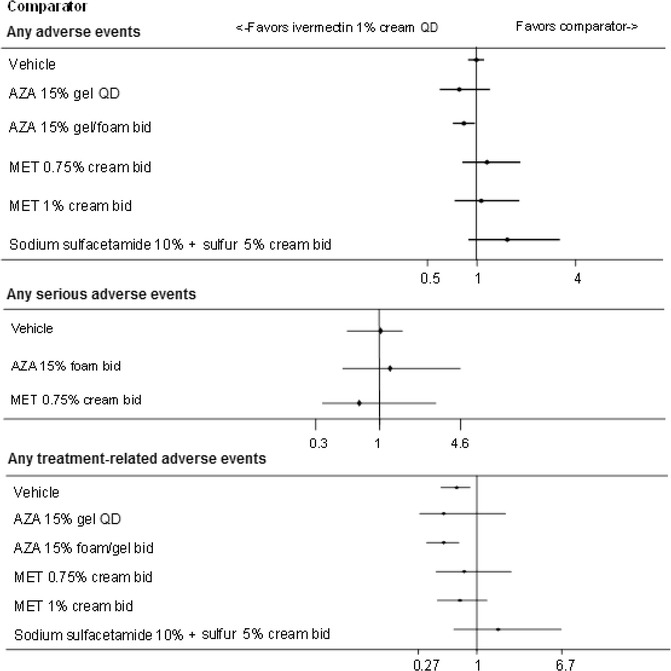
Results of MTC analyses between ivermectin 1 % cream QD and comparators for the incidence of any adverse events (*top*), serious adverse events (*middle*), and treatment-related adverse events (*bottom*) at 12 weeks. Risk ratios evaluate the probability of success (relieving rosacea) when using ivermectin 1 % cream QD, compared to other comparator treatments. A risk ratio >1 demonstrates a greater likelihood of success using ivermectin 1 % cream QD, a risk ratio credible interval that does not cross 1 demonstrates a significant difference between ivermectin 1 % cream QD and the comparator (positive values indicate superiority, negative values indicate inferiority). The comparison of ivermectin 1 % cream QD demonstrated significantly better results compared with azelaic acid 15 % gel/foam BID for any adverse events and with vehicle and azelaic acid 15 % gel/foam BID for any treatment-related adverse events. No comparator demonstrated significantly better results compared with ivermectin 1 % cream QD. Studies contributing to any adverse events: nine studies (Gold et al. 2014a; Stein et al. 2014; Galderma 2006; Tan et al. 2002; Thiboutot et al. 2003, 2008; Torok et al. 2005; NCT00617903 2013), any serious adverse events: four studies (Gold et al. 2014a, b; Galderma 2006; Draelos et al. 2015), any treatment-related adverse events: seven studies (Gold et al. 2014a; Stein et al. 2014; Galderma 2006; Tan et al. 2002; Thiboutot et al. 2008; Torok et al. 2005; Draelos et al. 2013). MTC results are derived from a fixed effects model. *AZA* azelaic acid, *bid* twice daily, *Crl* credible interval, *MET* metronidazole, *MTC* mixed treatment comparison, *QD* once daily, *RR* risk ratio

Thirteen studies contributed data for analysis of the safety endpoints at 12 weeks (Fig. 3). The MTC suggested that ivermectin 1 % cream QD was associated with a significantly lower risk of developing any AE compared with azelaic acid 15 % gel/foam BID [RR (95 % CrI): 0.83 (0.71–0.97)], with no significant difference between ivermectin 1 % cream QD and azelaic acid 15 % gel QD [0.78 (0.59–1.20)], metronidazole 0.75 % cream BID [1.15 (0.82–1.84)], metronidazole 1 % cream BID [1.06 (0.73–1.81)], sodium sulfacetamide 10 % in combination with sulfur 5 % cream BID [1.53 (0.89–3.20)], and vehicle [0.99 (0.88–1.10); Fig. 4]. Similarly, there were no significant differences in the incidence of SAEs reported with ivermectin 1 % cream QD compared with metronidazole 0.75 % cream BID [0.68 (0.34–2.88)], azelaic acid 15 % foam BID [1.22 (0.50–4.56)], or vehicle [1.02 (0.54–1.54); Fig. 4]. However, as only four studies contributed data for the analysis of SAEs at 12 weeks, care needs to be taken when interpreting the results (Gold et al. 2014a; Galderma 2006; Draelos et al. 2015).

There was a significantly lower risk of any TRAE with ivermectin 1 % cream QD compared with azelaic acid 15 % foam/gel BID [0.47 (0.32–0.67)] and vehicle [0.63 (0.45–0.86)], with no significant difference between ivermectin 1 % cream QD and all other comparators (Fig. 4).

**Fig. 5:**
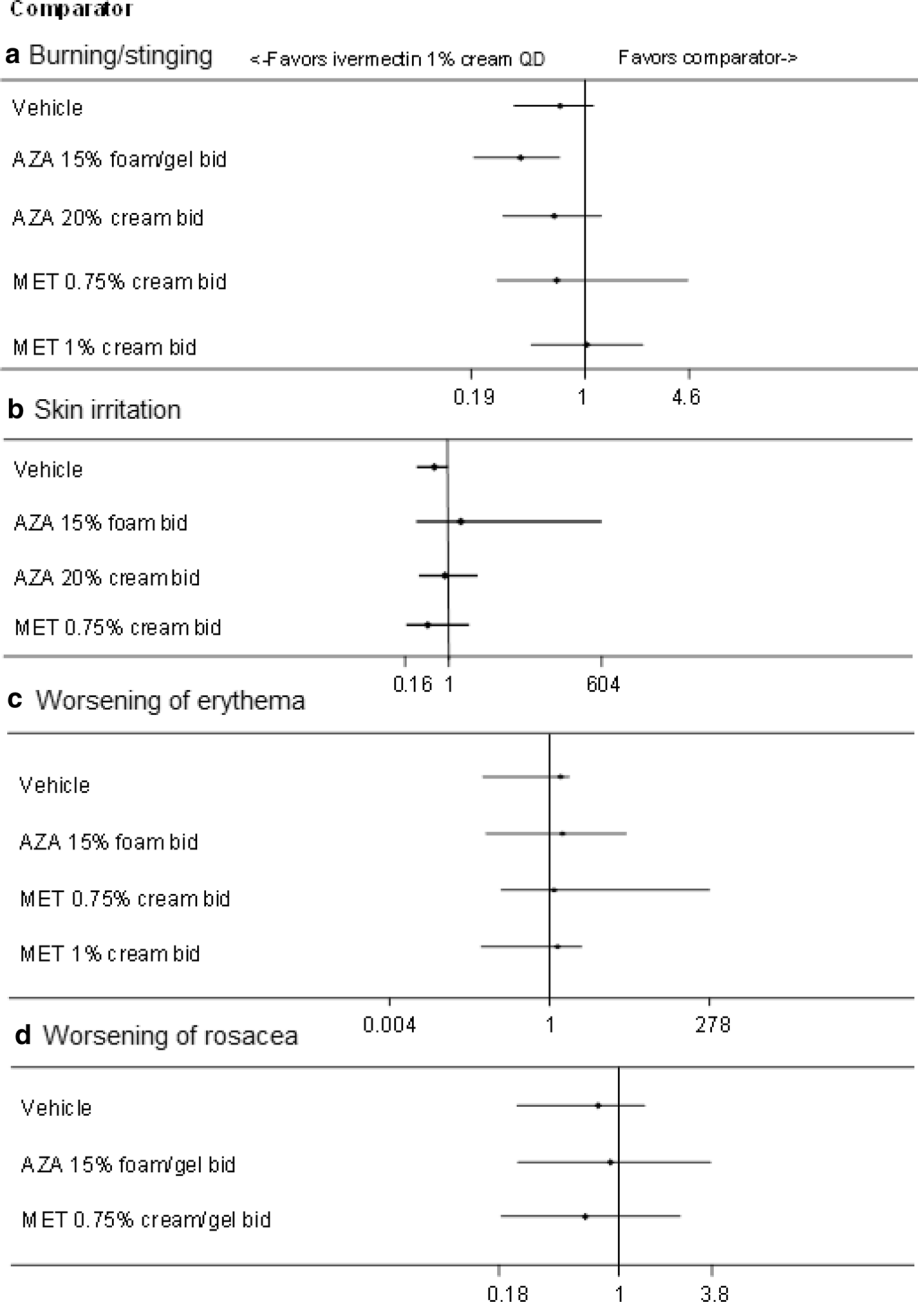
Results of MTC analyses between ivermectin 1 % cream QD and comparators for the incidence of specific adverse events at 12 weeks [burning/stinging (*A*), skin irritation (*B*), worsening or erythema (*C*), and worsening of rosacea (*D*)]. Risk ratios evaluate the probability of success (relieving rosacea) when using ivermectin 1 % cream QD, compared to other comparator treatments. A risk ratio >1 demonstrates a greater likelihood of success using ivermectin 1 % cream QD, a RR Crl that does not cross 1 demonstrates a significant difference between ivermectin 1 % cream QD and the comparator (positive values indicate superiority, negative values indicate inferiority). Studies contributing to *A*: six studies (Gold et al. 2014a; Stein et al. 2014; Tan et al. 2002; Galderma 2006; Bjerke et al. 1999; Draelos et al. 2013), *B*: four studies (Gold et al. 2014a; Stein et al. 2014; Galderma 2006; Bjerke et al. 1999), C: four studies (Tan et al. 2002; Galderma 2006; NCT00617903 2013; Draelos et al. 2013), *D*: four studies (Gold et al. 2014a; Galderma 2006; NCT00617903 2013; Draelos et al. 2013). MTC results are derived from a fixed effects model. *AZA* azelaic acid, *bid* twice daily, *Crl* credible interval, *MET* metronidazole, *MTC* mixed treatment comparison, *QD* once daily, *RR* risk ratio

Finally, ivermectin 1 % cream QD was associated with a statistically lower incidence of burning/stinging compared to azelaic acid 15 % gel/foam BID [0.39 (0.20–0.69)], and a lower incidence of skin irritation compared to vehicle [0.55 (0.26–0.96); Fig. 5]. The rates of burning/stinging, skin irritation, worsening of erythema, and worsening of rosacea were not statistically different across treatments for all other comparisons.

### Tolerability

The MTC of withdrawals due to AEs using data from 10 studies found no significant difference in the incidence between ivermectin 1 % cream QD and any of the comparators at 12 weeks (data not shown). The MTC of all-cause withdrawals (11 studies) suggested that ivermectin 1 % cream QD was associated with a significantly lower risk compared to pimecrolimus 1 % cream BID [RR 0.42 (95 % Crl 0.33–0.54)] with no significant difference compared with azelaic acid 15 % gel/foam BID, metronidazole 1 % cream BID, or vehicle (data not shown).

## Discussion

The aim of this systematic review was to build on the previous two Cochrane Collaboration reviews that primarily compared active treatments to placebo/vehicle (van Zuuren et al. 2011, 2015) and provide the first network meta-analysis of the efficacy, safety, and tolerability of a new topical treatment for the inflammatory lesions of rosacea, ivermectin 1 % cream QD, compared with other currently available topical treatments in patients with moderate-to-severe papulopustular rosacea. Although the previous Cochrane Collaboration systematic reviews pooled the direct evidence available and found evidence to support the use of topical azelaic acid, metronidazole, ivermectin, brimonidine, oral doxycycline, and oral tetracycline in the treatment of rosacea, since most of the trials available compare an active treatment to placebo/vehicle there was limited scope to compare across active treatments. Where there are a number of treatments available, it is important to compare the efficacy, safety, and tolerability across treatments, using quantitative analysis to perform indirect comparisons where direct head-to-head data are not available from the clinical trials.

The results of the quantitative analyses performed here suggest that ivermectin 1 % cream QD significantly increases the percentage reduction in inflammatory lesion count and increases the likelihood of success compared with azelaic acid 15 % cream BID and metronidazole 0.75 % gel BID up to 15 weeks of treatment. These results build on the conclusions from the most recent Cochrane Collaboration systematic review, which reported on the superior efficacy of ivermectin 1 % cream QD compared with metronidazole 0.75 % gel BID found in direct head-to-head trials (van Zuuren et al. 2015). Unsurprisingly, ivermectin 1 % cream QD was also significantly superior to vehicle for both success rates and inflammatory lesion counts at all time points, confirming that this is an effective topical treatment for patients with inflammatory lesions of rosacea.

The higher success rate and greater reduction in inflammatory lesions provided by ivermectin 1 % cream QD compared with other interventions may help to improve the QoL of patients with rosacea. Patients with rosacea have reported anxiety and depression attributable to their disease, which can be exacerbated by the side effects of current treatments (Huynh 2013). However, it is known that the QoL of patients with rosacea can be substantially improved during 3 months of effective treatment (Baldwin 2010). Indeed, during a head-to-head study of ivermectin 1 % cream QD versus metronidazole 0.75 % gel BID, patients receiving ivermectin 1 % cream QD reported a statistically significantly higher reduction in Dermatology Life Quality Index scores compared with patients receiving metronidazole 0.75 % gel BID, representing a greater improvement in QoL in alignment with the improved efficacy results observed (Taieb et al. 2015a). However, there remains a paucity of data on the humanistic burden of rosacea, and so further studies evaluating the effect of current topical treatments on QoL are needed. In addition, when interpreting the results of these indirect comparisons it is important to consider that conclusions are largely being drawn from a fixed effects model in a network where heterogeneity is difficult to assess. All conclusions based solely on indirect evidence could benefit from validation in the future.

Alongside the superior efficacy results, ivermectin 1 % cream QD demonstrates an acceptable safety profile with similar or lower rates of AEs compared with currently available topical treatments and vehicle. The only statistically significant differences across treatments favored ivermectin 1 % cream QD, where patients receiving ivermectin 1 % cream QD experienced significantly fewer AEs and TRAEs compared with azelaic acid 15 % cream BID. The favorable efficacy and safety profile of ivermectin 1 % cream QD could relate to an anti-inflammatory effect, which is a known property of this class of drug (van Zuuren et al. 2011). Although the mechanism of action of topical therapy for rosacea remains mostly unknown, it is hypothesized that ivermectin 1 % cream QD may reduce the ability of *Demodex folliculorum* mites to initiate inflammatory or specific immune reactions that lead to the symptoms of rosacea (Del Rosso et al. 2013a). Whereas current treatments target just one point in the immune-modulatory cascade, the potential ability of ivermectin 1 % cream QD to target both parasitic and inflammatory causes of rosacea may lead to a complete response on a more frequent basis and help to reduce the secondary symptoms of papulopustular rosacea.

This review provides evidence that ivermectin 1 % cream QD can provide sustained therapy for the inflammatory lesions of papulopustular rosacea up to 15 weeks. However, papulopustular rosacea is a chronic skin disease, and there remains a lack of data on the efficacy of long-term maintenance therapy with metronidazole or azelaic acid, as highlighted by the previous Cochrane review (van Zuuren et al. 2011). Indeed, three studies in this review had a study duration of just 6 weeks (Koch and Wilbrand 1999; Marks and Ellis 1971; Pye and Burton 1976), which allows demonstration of rapid improvement but not long-term clinical efficacy and safety. It is clear there is a need for studies investigating the long-term efficacy and safety of interventions for rosacea to assess treatment compliance, tolerability, and ability to maintain improvement/remission. To begin addressing this issue, the long-term efficacy and safety of ivermectin 1 % cream QD has been investigated in a 40-week extension period to two clinical trials comparing ivermectin 1 % cream QD with azelaic acid 15 % gel BID. Ivermectin 1 % cream QD demonstrated continued efficacy, with a higher proportion of patients with IGA score 0 or 1 (i.e. success) at the study endpoint compared to baseline than azelaic acid 15 % gel BID. Additionally, no subjects discontinued treatment with ivermectin 1 % cream QD due to an AE, and the incidence of TRAEs continued to be lower than with azelaic acid 15 % gel BID throughout the study (Stein et al. 2014). Overall, these two extension studies indicate that ivermectin 1 % cream QD is safe and effective for the treatment of the inflammatory lesions of rosacea up to 52 weeks, confirming and extending the time period for the conclusions of the quantitative analyses conducted here.

Additionally, the QD dosing of ivermectin 1 % cream is more convenient than the BID dosing of metronidazole 0.75 % gel and azelaic acid 15 % gel, and this may contribute to greater satisfaction with this treatment in addition to the superior efficacy (and in the case of azelaic acid 15 % gel, safety) observed in the analyses presented here. Indeed, patients treated with ivermectin 1 % cream QD report greater global improvement and satisfaction with the study drug compared with patients receiving metronidazole 0.75 % gel BID (Taieb et al. 2015a). Further, the ability to apply the ivermectin 1 % cream QD at night allows patients the option not to apply treatment in the morning, and instead apply only a sun protection factor (SPF). Such a dosing pattern may help improve the QoL and confidence of patients using SPF, since sun protection is important to calm skin irritation, redness and telangiectasia associated with rosacea (The National Rosacea Society 2015), Therefore, the dosing schedule and superior efficacy of ivermectin 1 % cream QD may lead to improvement in QoL and greater satisfaction with the study drug, which could ultimately lead to greater treatment compliance. This potential compliance benefit warrants further investigation given the clinical and economic benefits that are associated with compliance to treatment across diseases. Importantly, the superior results with ivermectin 1 % cream QD may reduce the need for patients to switch to systemic therapy after failure of first-line treatment. Although such a switch can be recommended in some cases (Del Rosso et al. 2013b), systemic treatments are associated with increased rates of AEs and antibiotic resistance (Goldgar et al. 2009). Therefore, ivermectin 1 % cream QD could prevent the negative consequences associated with progressing to systemic therapy.

When analyzing the results of this review, it is important to note that the studies contributing data for the efficacy outcomes of inflammatory lesion count demonstrated variability in clinical characteristics at baseline. Baseline inflammatory lesion counts were higher in studies investigating ivermectin 1 % cream QD (Gold et al. 2014a; Taieb et al. 2015a) compared with studies evaluating metronidazole 1 % cream QD or azelaic acid 15 % gel QD and BID. Although the higher baseline count with ivermectin 1 % cream QD may have resulted in a greater percentage change in inflammatory lesion count at each time point, the higher counts would also have made it more difficult to reach IGA 0 or 1 (and therefore achieve treatment success). Despite this additional difficulty in reaching success, ivermectin 1 % cream QD consistently demonstrates superior success rates compared with metronidazole 0.75 % cream BID and azelaic acid 15 % gel BID. Therefore, the greater efficacy of ivermectin 1 % cream QD as demonstrated across both success rate and inflammatory lesion count supports the effectiveness of ivermectin 1 % cream QD. In addition, the demonstrated ability to achieve success with ivermectin 1 % cream QD despite the high inflammatory lesion count at baseline suggests that this new intervention could be effective even for particularly severe cases of papulopustular rosacea, a population for which, for example, azelaic acid 15 % gel BID is not indicated. It is also important to recognize that success rate can be defined in a number of ways, which may impact on the data included in the analysis. Sensitivity analyses have been performed to examine the effect of different definitions of success (0 or 1 on a 5-point or 0 to 2 on a 7-point scale) on the results. No difference was demonstrated from the original results presented here, and so these findings can be considered robust and reliable irrespective of the IGA success rate definition applied.

Due to limited evidence, safety and tolerability outcomes were analyzed only at the 12-week time point, and so results should be interpreted with caution. However, the consistent results between the 40-week follow up study of ivermectin 1 % cream QD (Stein et al. 2014) and earlier results up to the 12-week time point (Gold et al. 2014a) suggest that the results reported here are likely to be similar to those seen during later weeks of therapy. Therefore, it is unlikely that this study failed to identify AEs of clinical significance to this patient population. This is supported by the lack of any further treatment-related discontinuations with ivermectin 1 % cream QD during the 40-week follow-up period (Stein et al. 2014).

Although previous reviews of the interventions for rosacea have been completed (van Zuuren et al. 2011, 2015), these reviews focused primarily on comparisons between an active comparator and placebo/vehicle as quantitative NMA were not conducted. In the current review, in order to provide sufficient data for analysis, evidence networks were prepared by combining different formulations of topical treatments, as long as the strength and dosing regimen of the drug did not vary. This approach allowed pooling of the data without introducing significant heterogeneity into the analysis, as it has been shown previously that the efficacy of metronidazole is similar regardless of the vehicle (cream, gel, or lotion) used to administer the treatment (Dahl et al. 2001; Maddin 1999). Such combinations allowed quantitative analysis for outcomes that otherwise could only have been presented qualitatively, providing data in addition to that available from direct head-to-head trials, and providing confirmation where head-to-head results were available. Although only 19 out of a potential 57 studies presented data in a form suitable for pooling, this represents 69 % of the total patient population due to the fact that many of the trials that could not be included in the NMA involved small patient populations.

It should be noted that a NMA was necessary because the available head-to-head evidence is limited for some comparisons. One key reason for performing an NMA is because there is an absence of direct evidence, and thus evaluation using indirect evidence is needed. However, inconsistency and heterogeneity between studies can only be assessed (and controlled for) to a limited extent, and as such additional studies verifying the conclusions drawn from solely indirect comparisons are warranted.

Given the potential superior efficacy of ivermectin 1 % cream QD compared to current topical treatments for up to 15 weeks, this treatment is a promising alternative option for patients with inflammatory lesions of rosacea, providing an additional therapy choice for physicians and dermatologists. Wider therapy choice is particularly crucial considering the lack of efficacy of metronidazole and azelaic acid in the most severe cases of papulopustular rosacea (van Zuuren et al. 2011), for which ivermectin 1 % cream QD has shown benefit.

## Conclusions

 Ivermectin 1 % cream QD appears to be a more effective, safer, and more tolerable topical treatment than other current treatments used to treat the inflammatory lesions of rosacea. Based on these results, ivermectin 1 % cream QD could provide physicians and dermatologists with an alternative first-line treatment option.

## Additional file


10.1186/s40064-016-2819-8 Supplementary Material supporting the network meta-analysis of the efficacy, safety, and tolerability of ivermectin compared with current topical treatments for the inflammatory lesions of rosacea.

## Abbreviations

AE: adverse event
BGR: Brooks–Gelman–Rubin
BID: twice-daily
CrI: credible intervals
CSR: clinical study report
DIC: deviance information criteria
D–L: DerSimonian and Laird
IGA: Investigator Global Assessment
M–H: Mantel–Haenszel
MTC: mixed treatment comparison
NICE: National Institute for Health and Care Excellence
NMA: network meta-analysis
NNT: number needed to treat
QD: once-daily
QoL: quality of life
RCT: randomized controlled trial
RR: risk ratio
SAE: serious adverse event
SD: standard deviation
SPF: sun protection factor
TRAE: treatment-related adverse event
