# Supplementary material for: The efficacy, safety, and tolerability of ivermectin compared with current topical treatments for the inflammatory lesions of rosacea: a network meta-analysis
Source: Springerplus. 2016 Jul 22;5(1):1151. doi: 10.1186/s40064-016-2819-8 (PMC4956638; doi:10.1186/s40064-016-2819-8)
Supplement: Supplementary file 1 — 10.1186/s40064-016-2819-8 Supplementary Material supporting the network meta-analysis of the efficacy, safety, and tolerability of ivermectin compared with current topical treatments for the inflammatory lesions of rosacea. [file 40064_2016_2819_MOESM1_ESM.docx]

**Supplementary Material**

**Article title**: The efficacy, safety, and tolerability of ivermectin compared with current topical treatments for the inflammatory lesions of rosacea: a network meta-analysis

**Journal name**: SpringerPlus

**Authors**: Kashif Siddiqui, Linda Stein Gold, Japinder Gill

**Corresponding author details**: PAREXEL Access Consulting, PAREXEL International. [Kashif.siddigui@parexel.com](mailto:Kashif.siddigui@parexel.com)

Systematic review search strategy

Table S1: Embase® and MEDLINE® search strategy in Embase® database for randomized controlled trials (searches conducted on June 19, 2014)

|  | Search strategy | Facet | Number of hits (June 19, 2014 | Number of hits (June 15, 2015 update) |
| --- | --- | --- | --- | --- |
| #1 | random*:ab,ti | Study design | 884 532 | 984 922 |
| #2 | factorial*:ab,ti |  | 23 407 | 25 677 |
| #3 | crossover:ab,ti OR 'cross over':ab,ti OR 'cross-over':ab,ti |  | 69 586 | 75 148 |
| #4 | 'placebo'/exp OR placebo |  | 329 592 | 351 867 |
| #5 | (doubl* OR singl*) NEAR/1 blind* |  | 205 788 | 220 727 |
| #6 | assign*:ab,ti OR allocat*:ab,ti |  | 319 303 | 352 960 |
| #7 | volunteer*:ab,ti OR 'volunteer'/exp |  | 188 675 | 201 339 |
| #8 | 'double blind procedure'/exp OR 'double blind procedure' |  | 115 338 | 121 819 |
| #9 | 'crossover procedure'/exp OR 'crossover procedure' |  | 39 070 | 42 966 |
| #10 | 'randomized controlled trial'/exp OR 'randomized controlled trial' |  | 402 228 | 451 615 |
| #11 | 'single blind procedure'/exp OR 'single blind procedure' |  | 18 135 | 19 909 |
| #12 | #1 OR #2 OR #3 OR #4 OR #5 OR #6 OR #7 OR #8 OR #9 OR #10 OR #11 |  | 1 550 853 | 1 704 898 |
| #13 | 'acne rosacea':ab,ti | Disease | 329 | 350 |
| #14 | 'rosacea'/exp OR 'rosacea' |  | 4521 | 4865 |
| #15 | 'rhinophyma'/exp OR rhinophyma |  | 4119 | 4423 |
| #16 | 'facial flushing':ab,ti |  | 738 | 800 |
| #17 | 'telangiectasis'/exp OR telangiectasis |  | 16 780 | 17 848 |
| #18 | 'pyoderma faciale' |  | 42 | 43 |
| #19 | 'rosacea fulminans' |  | 65 | 68 |
| #20 | morbihan* NEXT/1 disease |  | 23 | 27 |
| #21 | couperose |  | 17 | 17 |
| #22 | 'lupus' AND 'milaris' AND 'disseminata' AND 'facei' |  | 0 | 0 |
| #23 | 'granulomatous rosacea':ab,ti |  | 76 | 82 |
| #24 | ('facial' NEXT/3 ('telangiectasia' OR 'erythema' OR 'edema')):ab,ti |  | 1326 | 1471 |
| #25 | #13 OR #14 OR #15 OR #16 OR #17 OR #18 OR #19 Or #20 OR #21 OR #22 OR #23 OR #24 |  | 22 651 | 24 204 |
| #28 | #12 AND #25 | Study design AND Disease | 1445 | 1586 |
| #29 | (#12 AND #25) AND [9-2-2011]/sd NOT [19-06-2014]/sd  (limit from last Cochrane review search [2011] or from June 19, 2014 for update) |  | [480](http://embase.com/search/results?viewsearch=1) | 147 |

Abbreviations: ab: abstract; exp: expanded; sd: start date; ti: title

Network diagrams

**Figure S1:** Network diagrams for success rate at 3 weeks (A), 9 weeks (B), and 15 weeks (C)

**A**

**B**

**C**

AZA: Azelaic acid; bid: twice daily; IVE: Ivermectin; MET: Metronidazole; od: once daily

**Figure S2:** Network diagrams for percentage change in inflammatory lesion count at 3 weeks (A) and 9 weeks (B)

**A**

**B**

AZA: Azelaic acid; bid: twice daily; DOX: Doxycycline; IVE: Ivermectin; MET: Metronidazole; od: once daily

**Figure S3:** Network diagrams for incidence of burning/stinging (A), skin irritation (B), worsening of erythema (C), and worsening of rosacea (D) at 12 weeks

**A**

**
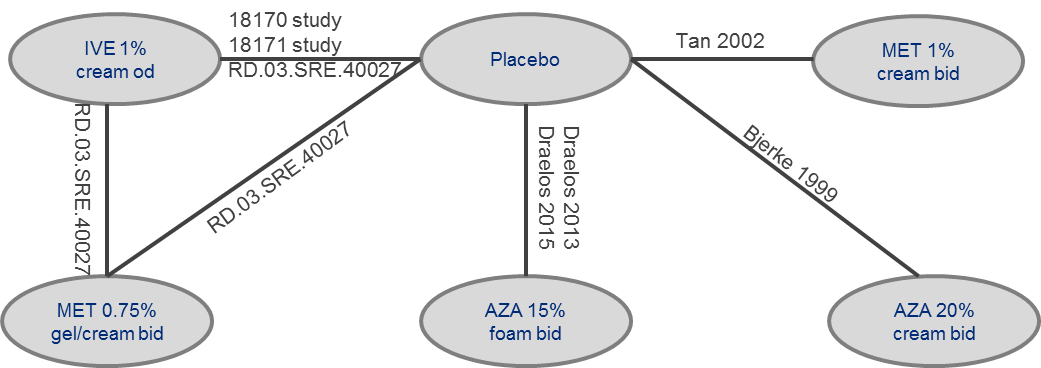
**

**B**

**
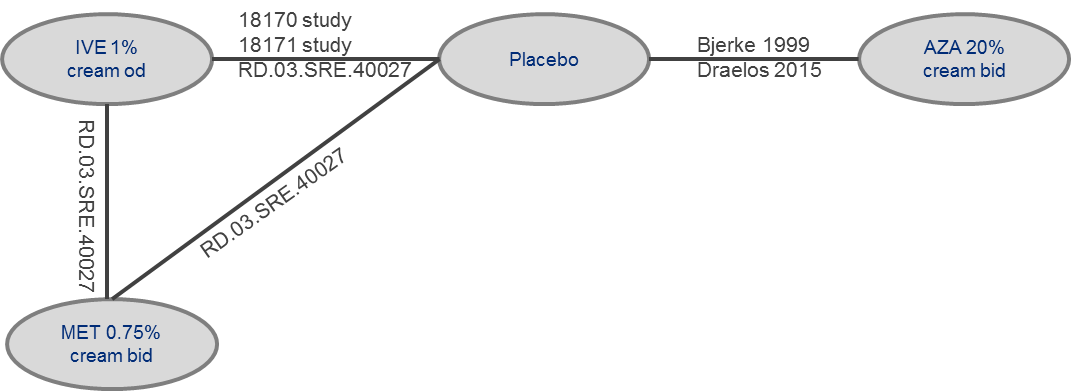
**

**C**

**
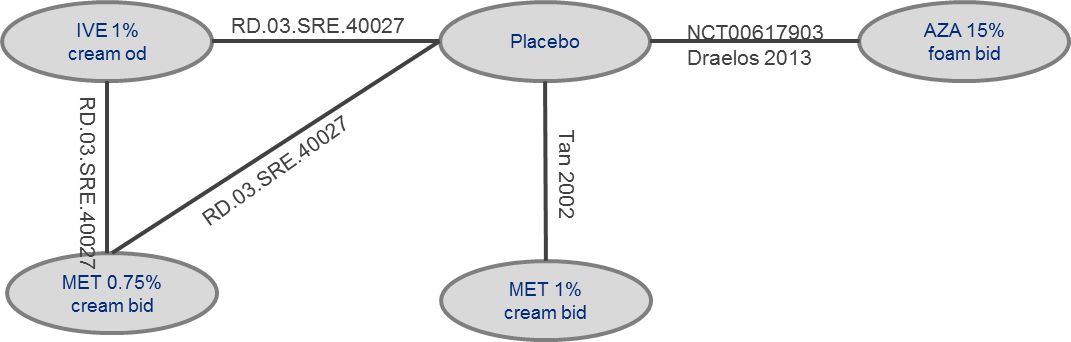
**

**D**

**
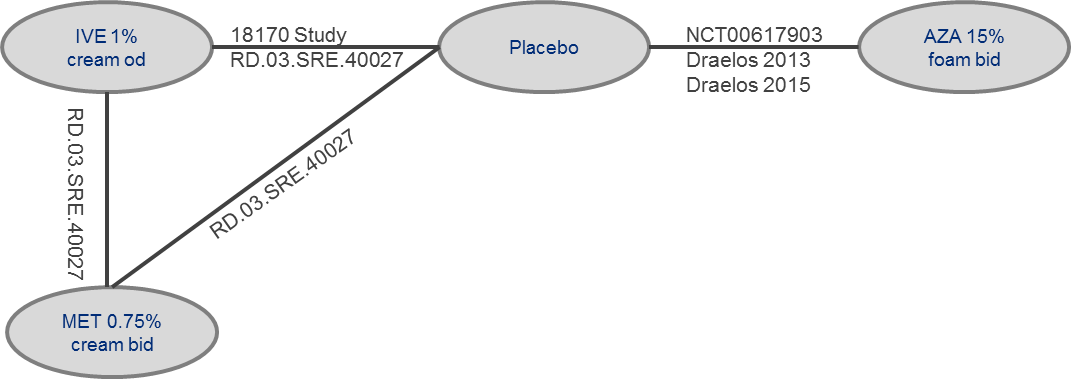
**

AZA: Azelaic acid; bid: twice daily; IVE: Ivermectin; MET: Metronidazole; od: once daily

**Figure S4:** Network diagrams for incidence of withdrawal due to any cause (A), and withdrawal due to adverse events (B) at 12 weeks

**A**

**
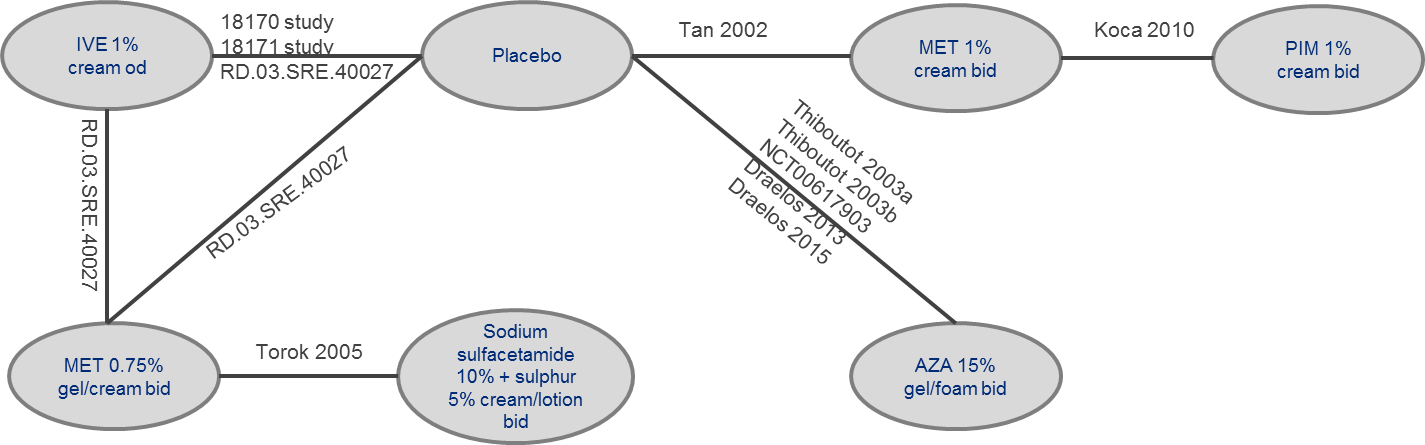
**

**B**

**
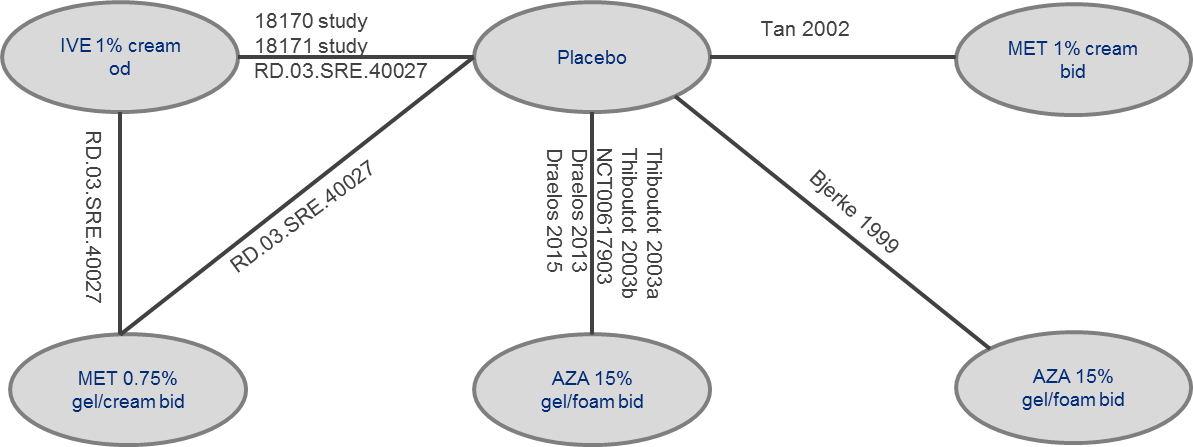
**

AZA: Azelaic acid; bid: twice daily; IVE: Ivermectin; MET: Metronidazole; od: once daily

Random effects model results

Table S2: Random effects results of an MTC of success rate for ivermectin 1% cream QD versus other available topical treatments and vehicle

| Comparator treatment | 3 weeks  6 studies | 9 weeks  7 studies | 12 weeks  12 studies | 15 weeks  3 studies |
| --- | --- | --- | --- | --- |
| RR (95% Crl) (vs. ivermectin 1% cream QD) | | | | |
| Azelaic acid 15% gel QD | - | - | 1.34 (0.95-2.58) | - |
| Azelaic acid 15% gel BID | 1.19 (0.62-2.48) | 1.36 (0.99-1.95) | 1.26 (1.08-1.48) | 0.99 (0.05-1.94) |
| Metronidazole 0.75% cream BID | 1.16 (0.75-3.09) | 1.10 (0.90-1.62) | 1.16 (1.00-1.38) | 1.34 (0.21-4.66) |
| Metronidazole 1% gel QD | 1.46 (0.62-8.26) | 1.23 (0.91-1.79) | 1.18 (0.92-1.80) | 1.03 (0.05-8.45) |
| Silica encapsulated benzoyl peroxide 1% gel QD | - | - | 1.10 (0.84-1.92) | - |
| Silica encapsulated benzoyl peroxide 5% gel QD | - | - | 0.95 (0.79-1.38) | - |
| Vehicle | 1.31 (0.76-1.72) | 1.50 (1.21-1.71) | 1.56 (1.39-1.69) | - |

Risk ratios evaluate the probability of success (relieving rosacea) when using ivermectin 1% cream QD, compared to other comparator treatments. A risk ratio >1 demonstrates a greater likelihood of success using ivermectin 1% cream QD, a RR Crl that does not cross 1 demonstrates a significant difference between ivermectin and the comparator (positive values indicate superiority, negative values indicate inferiority)

3 weeks: {Stein, 2014 7 /id;Galderma, 2014 69 /id;Wolf, 2006 81 /id;Draelos, 2013 44 /id;NCT00617903, 2013 65 /id;Taieb, 2015 8 /id}, 9 weeks: {Galderma, 2014 69 /id;Beutner, 2005 39 /id;Draelos, 2013 44 /id;NCT00617903, 2013 65 /id;Wolf, 2006 81 /id;Stein, 2014 7 /id;Taieb, 2015 8 /id}, 12 weeks: {Galderma, 2014 69 /id;Galderma, 2006 70 /id;Thiboutot, 2008 77 /id;Wolf, 2006 81 /id;NCT00617903, 2013 65 /id;Draelos, 2013 44 /id;Stein, 2014 7 /id;Thiboutot, 2003 76 /id;Leyden, 2014 83 /id;Draelos, 2015 84 /id;Taieb, 2015 8 /id}, 15 weeks: {Wolf, 2006 81 /id;Elewski, 2003 46 /id;Galderma, 2014 69 /id}

Abbreviations: BID: twice daily; Crl: credible interval; MTC: mixed treatment comparison; NNT: number needed-to-treat; QD: once daily; RR: risk ratio

Table S3: Random effects results of an MTC of percentage change in inflammatory lesion count between ivermectin 1% cream QD and comparators

| Comparator treatment | 3 weeks (Absolute difference, 95% Crl)  10 studies | 9 weeks (Absolute difference, 95% Crl)  11 studies | 12 weeks (Absolute difference, 95% Crl)  14 studies |
| --- | --- | --- | --- |
| Azelaic acid 15% gel QD | - | - | -15.47 (-30.17 to 0.22) |
| Azelaic acid 15% gel BID | -3.26 (-11.43 to 5.03) | -8.29 (-17.40 to -0.42) | -7.87 (-14.21 to -1.22) |
| Met 0.75% cream BID | -4.35 (-13.22 to 3.34) | -7.82 (-16.65 to 0.37) | -10.28 (-16.70 to -4.38) |
| Metronidazole 1% gel BID | 19.07 (-1.32 to 40.45) | 14.66 (-10.13 to 37.77) | 18.17 (-5.07 to 41.91) |
| Metronidazole 1% gel BID + doxycycline 40 mg QD | -9.37 (-37.45 to 20.00) | 6.23 (-23.05 to 34.20) | -0.15 (-27.30 to -28.67) |
| Sodium sulfacetamide 10%+sulfur 5% cream BID | - | 3.20 (-13.34 to 19.30) | -2.01 (-13.91 to -9.87) |
| Vehicle | -14.71 (-21.51 to -7.79) | -21.28 (-28.45 to -14.36) | -21.51 (-26.90 to -16.22) |

Negative values indicate a greater percentage reduction in the inflammatory lesion count with ivermectin 1% cream QD than with the comparator. A negative Crl that does not cross 0 indicates a significantly higher likelihood of patients using ivermectin 1% cream QD experiencing a greater reduction in inflammatory lesion count

3 weeks: {Galderma, 2014 69 /id;Fowler, 2007 47 /id;Tan, 2002 75 /id;Thiboutot, 2003 76 /id;Elewski, 2003 46 /id;NCT00617903, 2013 65 /id;Draelos, 2013 44 /id;Stein, 2014 7 /id;Taieb, 2015 8 /id}, 9 weeks: {Galderma, 2014 69 /id;Fowler, 2007 47 /id;Tan, 2002 75 /id;Thiboutot, 2003 78 /id;Elewski, 2003 46 /id;Torok, 2005 91 /id;NCT00617903, 2013 65 /id;Draelos, 2013 44 /id;Stein, 2014 7 /id;Taieb, 2015 8 /id}, 12 weeks: {Gold, 2014 48 /id;Galderma, 2006 70 /id;Galderma, 2014 69 /id;Torok, 2005 91 /id;Elewski, 2003 46 /id;Thiboutot, 2008 77 /id;Thiboutot, 2003 76 /id;Thiboutot, 2003 78 /id;NCT00617903, 2013 65 /id;Draelos, 2013 44 /id;Draelos, 2015 84 /id;Tan, 2002 75 /id;Fowler, 2007 47 /id}

MTC results are derived from a fixed effects model

Abbreviations: BID: twice daily; Crl: credible interval; MTC: mixed treatment comparison; QD: once daily

Fixed effects versus random effects model selection

An NMA can be performed using a fixed effect approach or a random effects approach. Guidance suggests that a random effects model should be used when included trials are relatively small (i.e. not mega-trials), and are heterogeneous in terms of patient population and design (Borenstein M). The deviance information criterion (DIC) was used to compare the fit of the random effects and fixed effects models in our analysis. The recommended methodology for comparing fit among a series of competing models is that a model whose DIC is at least three points lower than that of another model is deemed to have a better fit (Spiegelhalter DJ. A). Also taken into consideration when selecting the preferred model was the mean total residual deviance (compared against the number of fitted data points). In addition, the posterior distribution of the between studies standard deviation was investigated to ensure that it was updated from the prior distribution based on the available evidence. Where the prior distribution dominated, the fixed effect model was selected.

The number of included studies is small in many meta-analyses, leading to imprecision in estimation of the between-study heterogeneity variance when a random-effects model is used. It is therefore beneficial to perform a fully Bayesian random-effects meta-analysis and declare an appropriate informative prior distribution for heterogeneity (Turner et al).

In our analysis, there were no differences between DIC values and total residual differences between a fixed effects and a random effects model. Analysis results showed that there is large uncertainty and hence difficulty in estimating between studies SD with sufficient precision. The uncertainty around between studies SD was observed to be high and overwhelmed by vague priors. Please refer below for kernel density figures to match shapes for between SD from vague priors and actual prior distribution (U(0,2)) for success rate at 15 weeks (Supplementary Figure 1). Impact of priors was also tested by replacing vague priors with informative priors suggested by Turney et al 2012. The results of informative prior model were found to be concordant with fixed-effects model indicating the suitability of selection of the fixed effects model (Supplementary Table 3). Please refer to Supplementary Table 4 below for model parameters (DIC, totresdev, and between study SD).

**Table S4: Comparison of results from fixed effects, random effects vague priors, and random effects informative priors on variance**

| Comparator | NMA results | |  |
| --- | --- | --- | --- |
|  | Fixed effects model ES (95% CrI) | Random effects model ES (95% CrI)_Vague prior | Random effects model ES (95% CrI)_informative prior |
| Success rate at 3 weeks | | |  |
| Vehicle | **1.31 (1.07 to 1.54)** | 1.31 (0.76 to 1.72) | **1.31 (1.04, 1.55)** |
| AZA 15% gel bid | 1.18 (0.86 to 1.75) | 1.19 (0.62 to 2.48) | 1.19 (0.83, 1.80) |
| MET 0.75% cream bid | 1.16 (0.96 to 1.52) | 1.16 (0.75 to 3.09) | 1.16 (0.93, 1.62) |
| MET 1% gel od | 1.48 (0.83 to 4.22) | 1.46 (0.62 to 8.26) | 1.49 (0.81, 4.28) |
| Success rate at 9 weeks | | |  |
| Vehicle | **1.50 (1.37 to 1.61)** | **1.50 (1.21 to 1.71)** | **1.50 (1.35, 1.63)** |
| AZA 15% gel bid | **1.35 (1.13 to 1.65)** | 1.36 (0.99 to 1.95) | **1.36 (1.11, 1.71)** |
| MET 0.75% cream bid | **1.10 (1.02 to 1.21)** | 1.10 (0.90 to 1.62) | **1.10 (1, 1.28)** |
| MET 1% gel od | **1.23 (1.07 to 1.44)** | 1.23 (0.91 to 1.79) | **1.23 (1.04, 1.50)** |
| Success rate at 12 weeks | | |  |
| Vehicle | **1.56 (1.46 to 1.65)** | **1.56 (1.39 to 1.69)** | **1.56 (1.44, 1.66)** |
| AZA 15% gel bid | **1.25 (1.14 to 1.37)** | **1.26 (1.08 - 1.48)** | **1.26 (1.12, 1.41)** |
| AZA 15% gel od | 1.33 (0.99 to 2.20) | 1.34 (0.95 - 2.58) | 1.33 (0.97, 2.26) |
| MET 0.75% cream bid | **1.17 (1.08 to 1.29)** | **1.16 (1.00 - 1.38)** | **1.16 (1.04, 1.32)** |
| MET 1% gel od | 1.18 (0.98 - 1.56) | 1.18 (0.92 - 1.80) | 1.18 (0.96, 1.63) |
| BZP 1% gel od | 1.09 (0.86 - 1.78) | 1.10 (0.84 - 1.92) | 1.10 (0.86, 1.82) |
| BZP 5% gel od | 0.94 (0.81 - 1.29) | 0.95 (0.79 - 1.38) | 0.94 (0.81, 1.31) |
| Success rate at 15 weeks | | |  |
| AZA 15% gel bid | 1.00 (0.70 to 1.30) | 0.99 (0.05 to 1.94) | 1.00 (0.62,1.38) |
| MET 0.75% gel bid | **1.42 (1.19 to 1.71)** | 1.34 (0.21 to 4.66) | **1.41 (1.08, 1.84)** |
| MET 1% gel od | 1.06 (0.67 to 1.68) | 1.03 (0.05 to 8.45) | 1.06 (0.60, 1.83) |

**Table S5:** Comparison between fixed effects model and random effects model for MTC analyses

| **Time point** | **Contributing data points** | **Fixed effects** | **Random effects_ vague priors** | **Random effects_ Informative priors** |
| --- | --- | --- | --- | --- |
| **Success rate 3 weeks** | | | | |
| Deviance information criterion (DIC) | - | 72.210 | 74.017 | 72.631 |
| Mean (95% CI) total residual deviance | 12 | 10.25 (3.436 to 21.02) | 11.12 (3.856 to 21.99) | 10.43 (3.552 to 21.07) |
| Between-studies standard deviation # Mean (95% CI) | - | N/A | 0.5165 ( 0.015 to 1.734) | **0.1424 (0.0386 to 0.359)** |
| **Success rate 9 weeks** | | | | |
| Deviance information criterion (DIC) | - | 99.621 | 101.420 | 100.606 |
| Mean (95% CI) total residual deviance | 14 | 11.55 (4.386 to 22.47) | 12.3 (4.73 to 23.34) | 11.94 (4.574 to 23.0) |
| Between-studies standard deviation # Mean (95% CI) | - | N/A | 0.2815 ( 0.0083, 1.17) | **0.1289 (0.040, 0.424)** |
| **Success rate 12 weeks** | | | | |
| Deviance information criterion (DIC) | - | 182.019 | 182.779 | 181.887 |
| Mean (95% CI) total residual deviance | 26 | 29.68 (19.42 to 43.59) | 27.23 (15.19 to 42.2) | 27.69 (16.33 to 42.21) |
| Between-studies standard deviation # Mean (95% CI) | - | N/A | 0.2159 ( 0.0077 to 0.653) | **0.1372 (0.03961 to 0.3178)** |
| **Success rate 15 weeks** | | | | |
| Deviance information criterion (DIC) | - | 44.414 | 44.389 | 44.405 |
| Mean (95% CI) total residual deviance | 6 | 6.016 (1.237 to 14.5) | 6.017 (1.235 to 14.5) | 6.043 (1.237 to 14.53) |
| Between-studies standard deviation # Mean (95% CI) | - | N/A | 1.002 (0.04922, 1.953) | **0.157 (0.04007, 0.4237)** |

#Compared with vague prior U (0, 5)

**Figure S5:** Comparison of posterior between study SD and vague prior distribution (U (0,2)) for success rate at 15 weeks

Critical appraisal results

Table S6: Critical appraisal of included studies used in the presented analyses using the Scottish Intercollegiate Guidelines Network checklist

| SIGN section | Section 1: Internal validity | | | | | | | | | | Section 2: Overall assessment of the study | | | |
| --- | --- | --- | --- | --- | --- | --- | --- | --- | --- | --- | --- | --- | --- | --- |
| Question number^*^ | 1.1 | 1.2 | 1.3 | 1.4 | 1.5 | 1.6 | 1.7 | 1.8 | 1.9 | 1.10 | 2.1 | 2.2 | 2.3 | 2.4 |
| 18170 Study(Gold 2014a; Stein 2014) | Yes | Yes | Yes | Yes | Yes | Yes | Yes | Overall: 23.28% (Part A + Part B + Part C=159/683) Ivermectin: 22.39% (Part A + Part B + Part C=101) Placebo + azelaic acid: 25% (Part A + Part B + Part C=58) | Yes | Can't say | High quality (++) | Taking into account clinical considerations, evaluations of the methodology used, and the statistical power of the study, the effects observed in the study were due to the interventions in the trial | Yes | Ivermectin 1% cream QD was well-tolerated and safe, in particular, no notable difference was found between the ivermectin 1% cream QD and corresponding vehicle and azelaic acid 15% gel bid. The most frequent (>0.5% in any arm) adverse reactions were all skin disorders and were less frequent with ivermectin 1% cream QD than with the respective comparator. The success rate was statistically significantly higher in the ivermectin group at week 12, based on which it can be concluded that ivermectin is well-tolerated and effective in treatment of moderate‑to‑severe papulopustular rosacea |
| 18171 Study(Stein 2014; Gold 2014a) | Yes | Yes | Yes | Yes | Yes | Yes | Yes | Overall: 25.58% (Part A + Part B + Part C=176/688) Ivermectin: 23.09% (Part A + Part B + Part C=108/459) Placebo + azelaic acid: 30.57% (Part A + Part B + Part C=70/229) | Yes | Can't say | High quality (++) | Taking into account clinical considerations, evaluations of the methodology used, and the statistical power of the study, the effects observed in the study were due to the interventions in the trial | Yes | Ivermectin 1% cream QD was well-tolerated and safe, in particular, no notable difference was found between the ivermectin 1% cream QD and corresponding vehicle and azelaic acid 15% gel BID. The most frequent (>0.5% in any arm) adverse reactions were all skin disorders and were less frequent with ivermectin 1% cream QD than with the respective comparator. The success rate was statistically significantly higher in the ivermectin group at week 12, based on which it can be concluded that ivermectin is well-tolerated and effective in treatment of moderate‑to‑severe papulopustular rosacea |
| Beutner 2005(Beutner 2005) | Yes | Yes | Yes | Yes | Yes | Yes | Yes | Overall: 12% Metronidazole 1% gel QD: 10.2% Metronidazole 1% cream QD: 13% Placebo: 14.3% | Yes | Can't say | Acceptable (+) | Yes, the overall effect was due to the study intervention | Yes | This clinical study demonstrates that metronidazole 1% gel had a higher efficacy rate than its cream formulation and its vehicle and was equally well-tolerated. The results were significant in comparison to vehicle group but the p-value was not reported in comparison to the metronidazole 1% cream group, so we can say that metronidazole 1% gel is equally effective as metronidazole 1% cream when applied twice daily to treat rosacea |
| Bjerke 1999(Bjerke 1999) | Yes | Can't say | Can't say | Yes | Yes | Yes | Yes | Overall: 17.2% (114 evaluable patients) Treatment arm A (Azelaic acid 20% cream BID): not reported Treatment arm B (Placebo): not reported | No | Can't say | Acceptable (+) | Yes, the overall effect was due to the study intervention as there was no difference in the baseline characteristics between both the groups | Yes | In conclusion, azelaic acid 20% cream is effective and well-tolerated in the treatment of papulopustular rosacea. The results for decrease in inflammatory lesion count were statistically significant between the two arms |
| Draelos 2013(Draelos 2013) | Yes | Yes | Yes | Yes | Yes | Yes | Yes | Overall: 10.22% Azelaic acid 15% foam BID: 10.61 placebo: 9.85 | Yes | Can't say | High quality (++) | Yes, the overall effect was due to the study intervention as groups were treated equally and no other concomitant medication were allowed | Yes | The study demonstrated the efficacy and safety of a new azelaic acid foam in the treatment of patients with papulopustular rosacea when applied twice daily for 12 weeks. Azelaic acid demonstrated a statistically significant advantage over the vehicle in both therapeutic success rate (based on IGA) (p=0.017) and the nominal change in inflammatory lesion count from baseline (p=0.001) |
| Draelos 2015 | Yes | Can't say | Can't say | Yes | Yes | Yes | Yes | Overall: 14.8% (143 out of 961) Azelaic acid 15% foam BID: 13.2% (64 out of 484) Placebo: 16.1% (77 out of 477) | Yes | Can't say | Acceptable (+) | Yes, the overall effect was due to the study intervention as groups were treated equally | Yes | The authors concluded that this study supported the efficacy and safety of twice-daily azelaic acid foam in patients with papulopustular rosacea. Azelaic acid 15% foam demonstrated a statistically significant advantage over the vehicle in both primary measures of efficacy success rate (p<0.001) and change in inflammatory lesion count (p<0.001). |
| Elewski 2003(Elewski 2003) | Yes | Yes | Yes | Yes | Yes | Yes | Yes | Overall: 9.56% Azelaic acid 15% gel BID: 11.29% Metronidazole 0.75% gel BID: 7.87% | Yes | Can't say | High quality (++) | Yes, the overall effect was due to the study intervention as groups were treated equally and no other concomitant medication were allowed | Yes | The authors concluded that use of azelaic acid 15% gel BID for 15 weeks demonstrated significant superiority over using metronidazole 0.75% gel in improving principal signs of rosacea (inflammatory lesions and erythema). Standard deviation for mean inflammatory lesion counts was not reported |
| Fowler 2007(Fowler, Jr. 2007a; Fowler 2012) | Yes | Yes | Yes | Yes | Can't say | Can't say | Yes | Overall: 11.11% Metronidazole+ doxycycline: 16.67% Metronidazole+ Placebo: 5.52% | No | Can't say | Acceptable (+) | Can’t say, as information pertaining to the baseline characteristics, as well as confounding factors such as concomitant medication were not reported | Yes | The authors concluded that the combination of anti-inflammatory dose doxycycline and metronidazole 1% gel resulted in a faster reduction of inflammatory lesion count, when calculated at all interim and final data analysis. Also anti-inflammatory dose doxycycline from weeks 12 to 16 could sustain the beneficial effects of interventions given in week 1 to 12 and thus could be used to avoid long term use of topical or systemic antimicrobials. From the publication, it could not be ascertained the type of method of analysis used for both the safety and efficacy evaluations, thus bias in terms of reporting could not be ascertained |
| Koca 2010 | Yes | Yes | Can't say | No | No | Yes | Yes | Overall: 2.04% (1 out 49 patients) Metronidazole: 0% (0 out 24 patients) Pimecrolimus: 4.0% (1 out 25 patients) | Yes | Can't say | Acceptable (+) | Can't say; Since disease severity is subject to variation in accordance to many factors, the significant difference in disease duration between the two groups may raise an issue in considering whether the effects were due to the study medications or not | Yes | The author’s concluded that pimecrolimus cream was not more efficacious than metronidazole cream in the treatment of papulopustular rosacea. The results were not statistically significant for assessment of inflammatory lesion count at end of the study so both the treatments are equally effective to treat papulopustular rosacea. The study had a limitation of being an open-label trial and enrolled a very small number of patients. Also, there was statistically significant difference between the groups in terms of disease duration at baseline |
| Leyden 2014 | Yes | Can't say | Can't say | Yes | Yes | Yes | Yes | Not reported | Yes | Can't say | Acceptable (+) | Yes, the overall effect was due to the study intervention as groups were treated equally | Yes | The authors concluded that 1% and 5% silica encapsulated benzoyl peroxide were superior to vehicle in reducing papulopustular lesions (p=0.01) and (p=0.02). 5% silica encapsulated benzoyl peroxide was superior to vehicle for IGA (p=0.0013) |
| NCT00617903(NCT00617903 2013) | Yes | Yes | Can't say | Yes | Can't say | Can't say | Yes | Overall: 12.05% Azelaic acid 15% foam BID: 7.32% Placebo: 16.67% | Yes | Can't say | Acceptable (+) | Yes, the overall effect was due to the study intervention as groups were treated equally and no other concomitant medication was allowed | Yes | The authors’ conclusions about the study drug could not be ascertained from the NCT ID from where the trial data were extracted, no publication for the trial could be retrieved |
| RD.03.SRE.40027 study{Galderma, 2006 70 /id} | Yes | Yes | Yes | Yes | Yes | Yes | Yes | Overall: 7.77% (23 out of 296) Ivermectin 0.1% QD: 3.92%  (2 out of 51) Ivermectin 0.3% QD: 4.26%  (2 out of 47) Ivermectin 1% QD: 5.77% (3 out of 52) Ivermectin 0.1% BID: 10.42% (5 out of 48) Metronidazole 0.75% cream BID: 8.33%  (4 out of 48) Placebo: 14.0% (7 out of 50) | Yes | Can't say | High quality (++) | Yes; the overall effect is due to the study intervention | Yes | The authors concluded that both the QD and BID applications of ivermectin 1% cream were effective and safe in the papulopustular rosacea population. Since efficacy results between the two dosages were similar but compliance was enhanced by the ivermectin 1% QD application, this 1% dose was selected for further development |
| RD.03.SPR.40173 (ATTRACT) Study{Galderma, 2014 69 /id;Taieb, 2015 108 /id;Taieb, 2015 8 /id} | Yes | Can't say | Can't say | Yes | Yes | Yes | Yes | Overall: 6.24% (60 out of 962) Ivermectin: 6.69% (32 out of 478) Metronidazole: 5.79% (28 out of 484) | Yes | Can't say | High quality (++) | Yes; the overall effect is due to the study intervention | Yes | The author concluded that ivermectin 1% cream QD treatment resulted in a statistically significant extended remission (i.e. delayed time to first relapse, and increase in the number of treatment-free days) of rosacea when compared to metronidazole 0.75% BID in subjects who were successfully treated (IGA 0 [clear] or 1 [almost clear]) for 16 weeks. There was also a numerical trend in favor of ivermectin 1% cream QD for the relapse rates (62.7% and 68.4% in the ivermectin 1% group and metronidazole 0.75% group, respectively). It should be noted that the differences observed in favor of ivermectin 1% in Period B are presumably the consequence of the higher efficacy of ivermectin compared to metronidazole observed at the end of Period A, with a higher proportion of subjects with an IGA=0 in the ivermectin group (41.6% and 29.1% in ivermectin and metronidazole, respectively). Method of randomization and concealment allocation were not described, which could lead to bias in results |
| Tan 2002(Tan 2002) | Yes | Can't say | Can't say | Yes | Yes | Yes | Yes | Overall: 25.83%; Metronidazole 1% cream BID: 27.87%; Placebo: 23.73% | Yes | Can't say | Acceptable (+) | Details pertaining to method of randomization and allocation concealment, as well as statistical power of the study, were not reported. Taking into account clinical considerations, and an evaluation of the study methodology used, it appears that the overall effect is due to the study intervention | Yes | The authors concluded that the combined topical formulation of metronidazole 1% cream with sunscreen SPF 15 was effective and well-tolerated for the treatment of patients with moderate‑to‑severe rosacea. Metronidazole cream with sunscreen SPF 15 significantly decreased the clinical manifestations of rosacea (inflammatory papules and pustules, erythema, and telangiectasia). A plateau response was not attained by week 12 (end of study) for reduction in inflammatory lesions and reduction in erythema indicating that longer therapy may lead to greater response |
| Thiboutot 2003a(Thiboutot 2003a) | Yes | Yes | Can't say | Yes | Yes | Yes | Yes | Overall: 13.98% Azelaic acid 15% gel BID: 18.9% Placebo: 9.09% | Yes | Can't say | Acceptable (+) | Yes, the overall effect was due to the study intervention as no other medication was allowed that might influence outcome | Yes | The results demonstrate that azelaic acid 15% gel BID is an efficacious, safe, and well-tolerated topical treatment for moderate, papulopustular rosacea. The results were significant, so azelaic acid 15% gel BID is effective when applied to treat papulopustular rosacea |
| Thiboutot 2003b(Thiboutot 2003b) | Yes | Yes | Can't say | Yes | Yes | Yes | Yes | Overall: 11.64% Azelaic acid 15% gel BID: 11.24% Placebo: 12.04% | Yes | Can't say | Acceptable (+) | Yes, the overall effect was due to the study intervention as no other medication was allowed that might influence outcome | Yes | The results demonstrate that azelaic acid 15% gel BID is an efficacious, safe, and well-tolerated topical treatment for moderate, papulopustular rosacea. The results were significant so azelaic acid 15% gel BID is effective when applied to treat papulopustular rosacea |
| Thiboutot 2008(Thiboutot 2008) | Yes | Can't say | Yes | Yes | Yes | Yes | Yes | Overall: 4.35% | No | Can't say | Acceptable (+) | Yes, the overall effect was due to the study intervention as no other medication was allowed that might influence outcome | Yes | The authors concluded that azelaic acid 15% gel QD could be utilized as a safe, effective, and economical dosing option for the treatment of mild-to-moderate papulopustular rosacea. Once-daily dosing of azelaic acid 15% gel was well accepted by patients and offered considerable dosing flexibility and convenience for the patient as well as for the dermatologist. There were reporting biases in this study, as the results of one center were excluded, stating the results were not in conformity with the study protocol |
| Torok 2005(Torok 2005) | Yes | Yes | Yes | No | Can't say | Yes | Yes | Overall: 9.21% Sulfacetamide 10% and sulfur 5% cream BID: 13.3% Metronidazole 0.75% cream BID: 5.19% | Yes | Yes | Acceptable (+) | Yes, the overall effect was due to the study intervention, as groups were treated equally and no other concomitant medication were allowed | Yes | The authors concluded that in patients without sulpha drug allergies, sodium sulfacetamide 10% and sulfur 5% cream with sunscreens offers greater efficacy than metronidazole 0.75% cream and has the added benefit of sun protection |
| Wolf 2006(Wolf, Jr. 2006) | Yes | Yes | Can't say | Yes | Yes | Yes | Yes | Overall: 15% Metronidazole 1% gel QD: 17.07% Azelaic 15% gel BID: 12.82% | Yes | Can't say | Acceptable (+) | Yes, the overall effect was due to the study intervention as groups were treated equally | Yes | Both the treatments metronidazole 1% gel QD and azelaic acid 15% BID gel showed similar reductions in inflammatory lesion counts and high success rates in both global severity and erythema. On average, the efficacy of both the treatments was similar. There was no statistically significant difference between the groups, so metronidazole 1% gel QD and azelaic acid 15% gel BID are equally effective to treat papulopustular rosacea |

Sections relate to the 14 questions from the Scottish Intercollegiate Guidelines Network checklist for randomized controlled trials(SIGN Checklist 2015). “Yes” indicates the study meets the checklist criteria

BID: twice-daily; IGA: Investigator global assessment; QD: once-daily; SIGN: Scottish Intercollegiate Guidelines Network; SPF: sun protection factor

Other time point results

**Table S7:** Results of an MTC of success rate for ivermectin 1% cream QD versus other available topical treatments and vehicle at 3 weeks, 9 weeks, and 15 weeks

| Comparator treatment | 3 weeks  6 studies | 9 weeks  7 studies | 15 weeks  3 studies |
| --- | --- | --- | --- |
| **RR (95% Crl) (vs. ivermectin 1% cream QD)** | | | |
| Azelaic acid 15% gel QD | - | - | - |
| Azelaic acid 15% gel BID | 1.18 (0.86 to 1.75) | **1.35 (1.13 to 1.65)** | 1.00 (0.70 to 1.30) |
| Metronidazole 0.75% cream BID | 1.16 (0.96 to 1.52) | **1.10 (1.02 to 1.21)** | **1.42 (1.19 to 1.71)** |
| Metronidazole 1% gel QD | 1.48 (0.83 to 4.22) | **1.23 (1.07 to 1.44)** | 1.06 (0.67 to 1.68) |
| Silica encapsulated benzoyl peroxide 1% gel QD | **-** | - | - |
| Silica encapsulated benzoyl peroxide 5% gel QD | **-** | - | - |
| Vehicle | **1.31 (1.07 to 1.54)** | **1.50 (1.37 to 1.61)** | - |
| **NNT (95% Crl) (vs. vehicle)** |  |  |  |
| Azelaic acid 15% gel QD | - | - | - |
| Azelaic acid 15% gel BID | 33 (-638 to 629) | 21 (-265 to 297) | **7 (3 to 31)** |
| Metronidazole 0.75% cream BID | 30 (-573 to 602) | **7 (4 to 16)** | - |
| Metronidazole 1% gel QD | -25 (-387 to 372) | **13 (7 to 45)** | 7 (-70 to 80) |
| Silica encapsulated benzoyl peroxide 1% gel QD | - | **-** | - |
| Silica encapsulated benzoyl peroxide 5% gel QD | - | **-** | - |
| Ivermectin 1% cream QD | **21 (10 to 86)** | **5 (3 to 6)** | **7 (5 to 14)** |

Risk ratios evaluate the probability of success (relieving rosacea) when using ivermectin 1% cream QD, compared to other comparator treatments. A risk ratio >1 demonstrates a greater likelihood of success using ivermectin 1% cream QD, a RR Crl that does not cross 1 demonstrates a significant difference between ivermectin and the comparator (positive values indicate superiority, negative values indicate inferiority) (**indicated in bold**)

Lower estimates of NNT indicate a greater likelihood of patients achieving success with ivermectin 1% cream QD, as fewer patients need to be treated to achieve one success than with the comparator treatment. A positive Crl indicates a significantly greater likelihood of patients achieving success when using ivermectin 1% cream QD than when using the comparator (**indicated in bold**)

Studies contributing to 3 week analysis: {Gold, 2014 48 /id;Stein, 2014 7 /id;Galderma, 2014 69 /id;Wolf, 2006 81 /id;Draelos, 2013 44 /id;NCT00617903, 2013 65 /id}, 9 week analysis: {Galderma, 2014 69 /id;Beutner, 2005 39 /id;Draelos, 2013 44 /id;NCT00617903, 2013 65 /id;Wolf, 2006 81 /id;Stein, 2014 7 /id;Gold, 2014 48 /id}, 15 week analysis: {Wolf, 2006 81 /id;Elewski, 2003 46 /id;Galderma, 2014 69 /id}

MTC results are derived from a fixed effects model. At 15 weeks, ivermectin vs. vehicle data were limited, and so metronidazole 0.75% BID was used as the bridging comparator for NNT

Abbreviations: BID: twice daily; Crl: credible interval; MTC: mixed treatment comparison; NNT: number needed-to-treat; QD: once daily; RR: risk ratio

Table S8: Results of an MTC of percentage change in inflammatory lesion count between ivermectin 1% cream QD and comparators at 3 weeks and 9 weeks

| Comparator treatment | 3 weeks (Absolute difference, 95% Crl)  10 studies | 9 weeks (Absolute difference, 95% Crl)  11 studies |
| --- | --- | --- |
| Azelaic acid 15% gel QD | - | - |
| Azelaic acid 15% gel BID | -3.20 (-8.16 to 1.76) | **-7.63 (-11.66 to -3.53)** |
| Met 0.75% cream BID | -3.55 (-7.17 to 0.12) | **-7.60 (-11.12 to -4.07)** |
| Metronidazole 1% gel BID | **20.18 (2.15 to 38.05)** | 15.20 (-5.34 to 36.57) |
| Metronidazole 1% gel BID + doxycycline 40 mg QD | -8.80 (-34.63 to 17.21) | 6.56 (-17.69 to 31.33) |
| Sodium sulfacetamide 10%+sulfur 5% cream BID | - | 3.43 (-8.02 to 14.91) |
| Vehicle | **-14.71 (-18.49 to -10.85)** | **-20.94 (-24.68 to -17.18)** |

Negative values indicate a greater percentage reduction in the inflammatory lesion count with ivermectin 1% cream QD than with the comparator. A negative Crl that does not cross 0 indicates a significantly higher likelihood of patients using ivermectin 1% cream QD experiencing a greater reduction in inflammatory lesion count (significant differences between treatments indicated in **bold**)

Studies contributing to 3 week analysis: {Gold, 2014 48 /id;Galderma, 2014 69 /id;Fowler, 2007 47 /id;Tan, 2002 75 /id;Thiboutot, 2003 76 /id;Elewski, 2003 46 /id;NCT00617903, 2013 65 /id;Draelos, 2013 44 /id;Stein, 2014 7 /id}, 9 week analysis: {Galderma, 2014 69 /id;Fowler, 2007 47 /id;Tan, 2002 75 /id;Elewski, 2003 46 /id;Torok, 2005 91 /id;NCT00617903, 2013 65 /id;Draelos, 2013 44 /id;Stein, 2014 7 /id;Gold, 2014 48 /id;Thiboutot, 2003 76 /id}

MTC results are derived from a fixed effects model

Abbreviations: BID: twice daily; Crl: credible interval; MTC: mixed treatment comparison; QD: once daily

Matrix tables for success rate

**Table S9:** Results of an MTC of success rate at 3 weeks (fixed effects model)

|  | **Placebo** | **AZA 15% gel bid** | **MET 0.75% cream bid** | **MET 1% gel od** | **IVE 1% cream od** |
| --- | --- | --- | --- | --- | --- |
| **Placebo** | - | 0.9 (0.7 to 1.3) | 0.88 (0.68 to 1.29) | 1.13 (0.66 to 3.15) | 0.76 (0.65 to 0.93) |
| **AZA 15% gel bid** | 1.11 (0.77 to 1.43) | - | 0.98 (0.63 to 1.55) | 1.24 (0.75 to 3.1) | 0.84 (0.57 to 1.17) |
| **MET 0.75% cream bid** | 1.13 (0.78 to 1.47) | 1.02 (0.65 to 1.6) | - | 1.27 (0.65 to 3.66) | 0.86 (0.66 to 1.04) |
| **MET 1% gel od** | 0.89 (0.32 to 1.52) | 0.81 (0.32 to 1.33) | 0.79 (0.27 to 1.53) | - | 0.68 (0.24 to 1.21) |
| **IVE 1% cream od** | 1.31 (1.07 to 1.54) | 1.18 (0.86 to 1.75) | 1.16 (0.96 to 1.52) | 1.48 (0.83 to 4.22) | - |

**Table S10:** Results of an MTC of success rate at 3 weeks (random effects model)

|  | **Placebo** | **AZA 15% gel bid** | **MET 0.75% cream bid** | **MET 1% gel od** | **IVE 1% cream od** |
| --- | --- | --- | --- | --- | --- |
| **Placebo** | - | 0.91 (0.61 to 1.85) | 0.89 (0.55 to 3.01) | 1.12 (0.57 to 6.43) | 0.76 (0.58 to 1.32) |
| **AZA 15% gel bid** | 1.1 (0.54 to 1.63) | - | 0.98 (0.42 to 3.46) | 1.22 (0.64 to 5.2) | 0.84 (0.4 to 1.62) |
| **MET 0.75% cream bid** | 1.13 (0.33 to 1.81) | 1.02 (0.29 to 2.35) | - | 1.25 (0.33 to 7.07) | 0.86 (0.32 to 1.34) |
| **MET 1% gel od** | 0.89 (0.16 to 1.77) | 0.82 (0.19 to 1.57) | 0.8 (0.14 to 3.03) | - | 0.69 (0.12 to 1.6) |
| **IVE 1% cream od** | 1.31 (0.76 to 1.72) | 1.19 (0.62 to 2.48) | 1.16 (0.75 to 3.09) | 1.46 (0.62 to 8.26) | - |

**Table S11:** Results of an MTC of success rate at 9 weeks (fixed effects model)

|  | **Placebo** | **AZA 15% gel bid** | **MET 0.75% cream bid** | **MET 1% gel od** | **IVE 1% cream od** |
| --- | --- | --- | --- | --- | --- |
| **Placebo** | - | 0.9 (0.77 to 1.09) | 0.73 (0.65 to 0.85) | 0.82 (0.74 to 0.94) | 0.67 (0.62 to 0.73) |
| **AZA 15% gel bid** | 1.11 (0.92 to 1.29) | - | 0.81 (0.65 to 1.01) | 0.91 (0.76 to 1.09) | 0.74 (0.61 to 0.88) |
| **MET 0.75% cream bid** | 1.36 (1.18 to 1.53) | 1.23 (0.99 to 1.53) | - | 1.12 (0.93 to 1.34) | 0.91 (0.83 to 0.98) |
| **MET 1% gel od** | 1.21 (1.06 to 1.36) | 1.09 (0.92 to 1.32) | 0.89 (0.75 to 1.07) | - | 0.81 (0.7 to 0.93) |
| **IVE 1% cream od** | 1.5 (1.37 to 1.61) | 1.35 (1.13 to 1.65) | 1.10 (1.02 to 1.21) | 1.23 (1.07 to 1.44) | - |

**Table S12:** Results of an MTC of success rate at 9 weeks (random effects model)

|  | **Placebo** | **AZA 15% gel bid** | **MET 0.75% cream bid** | **MET 1% gel od** | **IVE 1% cream od** |
| --- | --- | --- | --- | --- | --- |
| **Placebo** | - | 0.91 (0.72 to 1.3) | 0.73 (0.58 to 1.2) | 0.83 (0.66 to 1.19) | 0.67 (0.59 to 0.83) |
| **AZA 15% gel bid** | 1.1 (0.77 to 1.4) | - | 0.81 (0.53 to 1.38) | 0.91 (0.66 to 1.26) | 0.74 (0.51 to 1.01) |
| **MET 0.75% cream bid** | 1.36 (0.83 to 1.74) | 1.24 (0.73 to 1.88) | - | 1.12 (0.67 to 1.71) | 0.91 (0.62 to 1.12) |
| **MET 1% gel od** | 1.21 (0.84 to 1.52) | 1.1 (0.79 to 1.51) | 0.89 (0.58 to 1.5) | - | 0.81 (0.56 to 1.1) |
| **IVE 1% cream od** | 1.50 (1.21 to 1.71) | 1.36 (0.99 to 1.95) | 1.10 (0.9 to 1.62) | 1.23 (0.91 to 1.79) | - |

**Table S13:** Results of an MTC of success rate at 12 weeks (fixed effects model)

|  | **Placebo** | **AZA 15% gel/foam bid** | **AZA 15% gel od** | **MET 0.75% cream bid** | **MET 1% gel od** | **BZP 1% gel od** | **BZP 5% gel od** | **IVE 1% cream od** |
| --- | --- | --- | --- | --- | --- | --- | --- | --- |
| **Placebo** | - | 0.8 (0.75 to 0.86) | 0.85 (0.64 to 1.4) | 0.75 (0.67 to 0.85) | 0.75 (0.63 to 0.99) | 0.7 (0.56 to 1.14) | 0.6 (0.53 to 0.82) | 0.64 (0.61 to 0.68) |
| **AZA 15% gel/foam bid** | 1.25 (1.16 to 1.33) | - | 1.06 (0.8 to 1.73) | 0.93 (0.82 to 1.08) | 0.94 (0.79 to 1.22) | 0.87 (0.69 to 1.42) | 0.75 (0.64 to 1.03) | 0.8 (0.73 to 0.87) |
| **AZA 15% gel od** | 1.17 (0.71 to 1.56) | 0.94 (0.58 to 1.25) | - | 0.88 (0.53 to 1.21) | 0.89 (0.53 to 1.3) | 0.83 (0.48 to 1.43) | 0.71 (0.43 to 1.07) | 0.75 (0.46 to 1.01) |
| **MET 0.75% cream bid** | 1.34 (1.17 to 1.49) | 1.07 (0.93 to 1.22) | 1.14 (0.83 to 1.89) | - | 1.01 (0.81 to 1.35) | 0.94 (0.72 to 1.53) | 0.81 (0.67 to 1.11) | 0.86 (0.78 to 0.92) |
| **MET 1% gel od** | 1.33 (1.01 to 1.58) | 1.07 (0.82 to 1.27) | 1.13 (0.77 to 1.88) | 0.99 (0.74 to 1.24) | - | 0.93 (0.65 to 1.55) | 0.8 (0.59 to 1.14) | 0.85 (0.64 to 1.03) |
| **BZP 1% gel od** | 1.43 (0.88 to 1.79) | 1.15 (0.7 to 1.46) | 1.21 (0.7 to 2.09) | 1.07 (0.65 to 1.4) | 1.08 (0.64 to 1.54) | - | 0.87 (0.6 to 1.08) | 0.91 (0.56 to 1.16) |
| **BZP 5% gel od** | 1.66 (1.22 to 1.89) | 1.33 (0.97 to 1.55) | 1.4 (0.93 to 2.35) | 1.24 (0.9 to 1.5) | 1.25 (0.88 to 1.69) | 1.15 (0.92 to 1.68) | - | 1.06 (0.78 to 1.23) |
| **IVE 1% cream od** | 1.56 (1.46 to 1.65) | 1.25 (1.14 to 1.37) | 1.33 (0.99 to 2.20) | 1.17 (1.08 to 1.29) | 1.18 (0.98 to 1.56) | 1.09 (0.86 to 1.78) | 0.94 (0.81 to 1.29) | - |

**Table S14:** Results of an MTC of success rate at 12 weeks (random effects model)

|  | **Placebo** | **AZA 15% gel/foam bid** | **AZA 15% gel od** | **MET 0.75% cream bid** | **MET 1% gel od** | **BZP 1% gel od** | **BZP 5% gel od** | **IVE 1% cream od** |
| --- | --- | --- | --- | --- | --- | --- | --- | --- |
| **Placebo** | - | 0.81 (0.73 to 0.94) | 0.86 (0.62 to 1.65) | 0.74 (0.63 to 0.92) | 0.76 (0.61 to 1.15) | 0.7 (0.55 to 1.23) | 0.61 (0.53 to 0.88) | 0.64 (0.59 to 0.72) |
| **AZA 15% gel/foam bid** | 1.24 (1.07 to 1.38) | - | 1.06 (0.78 to 1.98) | 0.92 (0.73 to 1.16) | 0.94 (0.75 to 1.37) | 0.87 (0.66 to 1.53) | 0.76 (0.61 to 1.1) | 0.79 (0.67 to 0.92) |
| **AZA 15% gel od** | 1.17 (0.61 to 1.6) | 0.94 (0.51 to 1.29) | - | 0.86 (0.45 to 1.27) | 0.89 (0.45 to 1.44) | 0.83 (0.42 to 1.56) | 0.72 (0.37 to 1.14) | 0.75 (0.39 to 1.05) |
| **MET 0.75% cream bid** | 1.35 (1.08 to 1.6) | 1.09 (0.86 to 1.37) | 1.16 (0.79 to 2.23) | - | 1.03 (0.75 to 1.6) | 0.95 (0.68 to 1.68) | 0.83 (0.63 to 1.22) | 0.86 (0.73 to 1) |
| **MET 1% gel od** | 1.32 (0.87 to 1.65) | 1.07 (0.73 to 1.33) | 1.13 (0.7 to 2.2) | 0.98 (0.63 to 1.33) | - | 0.93 (0.58 to 1.65) | 0.81 (0.51 to 1.22) | 0.85 (0.55 to 1.09) |
| **BZP 1% gel od** | 1.42 (0.82 to 1.82) | 1.14 (0.65 to 1.53) | 1.21 (0.64 to 2.41) | 1.05 (0.6 to 1.47) | 1.07 (0.61 to 1.74) | - | 0.88 (0.56 to 1.13) | 0.91 (0.52 to 1.2) |
| **BZP 5% gel od** | 1.65 (1.14 to 1.9) | 1.32 (0.91 to 1.63) | 1.39 (0.87 to 2.68) | 1.21 (0.82 to 1.59) | 1.23 (0.82 to 1.94) | 1.14 (0.88 to 1.77) | - | 1.05 (0.72 to 1.27) |
| **IVE 1% cream od** | 1.56 (1.39 to 1.69) | 1.26 (1.08 to 1.48) | 1.34 (0.95 to 2.58) | 1.16 (1 to 1.38) | 1.18 (0.92 to 1.8) | 1.1 (0.84 to 1.92) | 0.95 (0.79 to 1.38) | - |

**Table S15:** Results of an MTC of success rate at 15 weeks (fixed effects model)

|  | **AZA 15% gel bid** | **MET 0.75% gel bid** | **MET 1% gel od** | **IVE 1% cream od** |
| --- | --- | --- | --- | --- |
| **AZA 15% gel bid** | - | 1.43 (1.05 to 2.07) | 1.06 (0.8 to 1.55) | 1 (0.77 to 1.43) |
| **MET 0.75% gel bid** | 0.7 (0.48 to 0.95) | - | 0.75 (0.47 to 1.21) | 0.71 (0.59 to 0.84) |
| **MET 1% gel od** | 0.94 (0.64 to 1.25) | 1.34 (0.83 to 2.13) | - | 0.95 (0.6 to 1.49) |
| **IVE 1% cream od** | 1.00 (0.7 to 1.3) | 1.42 (1.19 to 1.71) | 1.06 (0.67 to 1.68) | - |

**Table S16:** Results of an MTC of success rate at 12 weeks (random effects model)

|  | **AZA 15% gel bid** | **MET 0.75% gel bid** | **MET 1% gel od** | **IVE 1% cream od** |
| --- | --- | --- | --- | --- |
| **AZA 15% gel bid** | - | 1.44 (0.57 to 12.64) | 1.06 (0.54 to 8.08) | 1.01 (0.51 to 20.16) |
| **MET 0.75% gel bid** | 0.7 (0.08 to 1.74) | - | 0.75 (0.08 to 6.46) | 0.75 (0.21 to 4.69) |
| **MET 1% gel od** | 0.95 (0.12 to 1.85) | 1.33 (0.16 to 12.48) | - | 0.97 (0.12 to 18.42) |
| **IVE 1% cream od** | 0.99 (0.05 to 1.94) | 1.34 (0.21 to 4.66) | 1.03 (0.05 to 8.45) | - |
